# Breadth of SARS-CoV-2 neutralization and protection induced by a nanoparticle vaccine

**DOI:** 10.1038/s41467-022-33985-4

**Published:** 2022-10-23

**Authors:** Dapeng Li, David R. Martinez, Alexandra Schäfer, Haiyan Chen, Maggie Barr, Laura L. Sutherland, Esther Lee, Robert Parks, Dieter Mielke, Whitney Edwards, Amanda Newman, Kevin W. Bock, Mahnaz Minai, Bianca M. Nagata, Matthew Gagne, Daniel C. Douek, C. Todd DeMarco, Thomas N. Denny, Thomas H. Oguin, Alecia Brown, Wes Rountree, Yunfei Wang, Katayoun Mansouri, Robert J. Edwards, Guido Ferrari, Gregory D. Sempowski, Amanda Eaton, Juanjie Tang, Derek W. Cain, Sampa Santra, Norbert Pardi, Drew Weissman, Mark A. Tomai, Christopher B. Fox, Ian N. Moore, Hanne Andersen, Mark G. Lewis, Hana Golding, Robert Seder, Surender Khurana, Ralph S. Baric, David C. Montefiori, Kevin O. Saunders, Barton F. Haynes

**Affiliations:** 1 Duke Human Vaccine Institute, Duke University School of Medicine, Durham, NC 27710 USA; 2 Department of Medicine, Duke University School of Medicine, Durham, NC 27710 USA; 3 Department of Epidemiology, University of North Carolina at Chapel Hill, Chapel Hill, NC 27599 USA; 4 Department of Surgery, Duke University School of Medicine, Durham, NC 27710 USA; 5 Infectious Disease Pathogenesis Section, Comparative Medicine Branch, National Institute of Allergy and Infectious Diseases, National Institutes of Health, Bethesda, MD 20814 USA; 6 Vaccine Research Center, National Institute of Allergy and Infectious Diseases, National Institutes of Health, Bethesda, MD 20814 USA; 7 Division of Viral Products, Center for Biologics Evaluation and Research (CBER), Food and Drug Administration, Silver Spring, MD 20871 USA; 8 Beth Israel Deaconess Medical Center, Boston, MA 02215 USA; 9 Department of Microbiology, University of Pennsylvania, Philadelphia, PA 19104 USA; 10 Department of Medicine, University of Pennsylvania, Philadelphia, PA 19104 USA; 11 Corporate Research Materials Lab, 3M Company, St Paul, MN 55144 USA; 12 Infectious Disease Research Institute, Seattle, WA 98104 USA; 13 BIOQUAL, Rockville, MD 20850 USA; 14 Department of Immunology, Duke University School of Medicine, Durham, NC 27710 USA; 15 Department of Molecular Genetics and Microbiology, Duke University School of Medicine, Durham, NC 27710 USA

**Keywords:** Conjugate vaccines, RNA vaccines, SARS-CoV-2

## Abstract

Coronavirus vaccines that are highly effective against current and anticipated SARS-CoV-2 variants are needed to control COVID-19. We previously reported a receptor-binding domain (RBD)-sortase A-conjugated ferritin nanoparticle (scNP) vaccine that induced neutralizing antibodies against SARS-CoV-2 and pre-emergent sarbecoviruses and protected non-human primates (NHPs) from SARS-CoV-2 WA-1 infection. Here, we find the RBD-scNP induced neutralizing antibodies in NHPs against pseudoviruses of SARS-CoV and SARS-CoV-2 variants including 614G, Beta, Delta, Omicron BA.1, BA.2, BA.2.12.1, and BA.4/BA.5, and a designed variant with escape mutations, PMS20. Adjuvant studies demonstrate variant neutralization titers are highest with 3M-052-aqueous formulation (AF). Immunization twice with RBD-scNPs protect NHPs from SARS-CoV-2 WA-1, Beta, and Delta variant challenge, and protect mice from challenges of SARS-CoV-2 Beta variant and two other heterologous sarbecoviruses. These results demonstrate the ability of RBD-scNPs to induce broad neutralization of SARS-CoV-2 variants and to protect animals from multiple different SARS-related viruses. Such a vaccine could provide broad immunity to SARS-CoV-2 variants.

## Abstract (web summary)

The authors have previously demonstrated the neutralising capacity of their nanoparticle vaccine, as well as showing protection of non-human primates from SARS-CoV-2 WA-1 infection. In this work, they investigate the ability of their vaccine candidate to neutralise SARS-CoV-2 variants of concern, and protect animals from other sarbecoviruses.

## Introduction

Despite the initial success of approved COVID-19 mRNA vaccines^1–3^, additional broadly protective vaccines are needed to combat breakthrough infections caused by waning immunity to emerging SARS-CoV-2 variants^4^. New vaccines can either be developed against emerging variants, or vaccines with the original SARS-CoV-2 strain antigens can be made more potent, inducing very high neutralizing titers such that variants that escape will still be potently neutralized by induced antibody titers.

The mRNA-1273 and the BNT162b2 COVID-19 vaccines, while showing large reductions in antibody-mediated neutralization against SARS-CoV-2 B.1.351 (Beta) and B.1.1.529 (Omicron) variants, continue to provide significant protection from serious COVID-19 disease, hospitalization, and death^4,5^. Likely arising from immunocompromised individuals in South Africa, the spike proteins of Omicron variants contain ~30 mutations compared to the WA-1 strain, and continue to evolve into Omicron sublineages^6,7^. While less pathogenic than Delta and other SARS-CoV-2 variants, the enhanced transmissibility of Omicron sublineage BA.2^8^, coupled with the sheer number of resulting cases, has resulted in a higher absolute number of COVID-19 patients compared to previous variant infections, thus providing a continued burden on global health care systems. The BA.2 variant has now outcompeted BA.1 and accounts for 95% of recent COVID-19 transmissions (http://www.gisaid.org/hcov19-variants). New Omicron sublineages (BA.2.12.1, BA.4, BA.5) have emerged with L453 and F486 mutations and remarkably, have greater transmissibility than BA.2^9^.

We previously reported a receptor-binding domain (RBD)-based, sortase A-conjugated nanoparticle (RBD-scNP) vaccine formulated with the TLR7/8 agonist 3M-052-aqueous formulation (AF) plus Alum, that elicited cross-neutralizing antibody responses against SARS-CoV-2 and other sarbecoviruses, and protected against the SARS-CoV-2 WA-1 strain in non-human primates (NHPs)^10^. Here, we found RBD-scNPs induced antibodies that neutralized SARS-CoV and SARS-CoV-2 variants including Beta, Omicron BA.1, BA.2, BA.2.12.1, BA.4 and BA.5 as well as a designed neutralization escape variant, PMS20^11^. Formulating RBD-scNP with Alum, 3M-052-AF, or 3M-052-Alum each protected macaques from WA-1 challenge. Importantly, the 3M-052-AF/ RBD-scNP formulation was optimal for induction of neutralization titers to Omicron variants. In addition, we found that RBD-, N-terminal domain (NTD)- and stabilized spike-2P (S2P)-scNPs each protected comparably in the upper and lower airways from WA-1, but boosting with the NTD-scNP protected less well than RBD-scNP or S2P-scNP. Finally, two doses of RBD-scNP immunization protected against Beta and Delta variant challenges in macaques, and protected in mouse models against challenges with the SARS-CoV-2 Beta variant and other sarbecoviruses.

## Results

### Optimization of adjuvant formulation for RBD-scNP induction of SARS-CoV-2 Omicron variant neutralizing antibodies

**Fig. 1:**
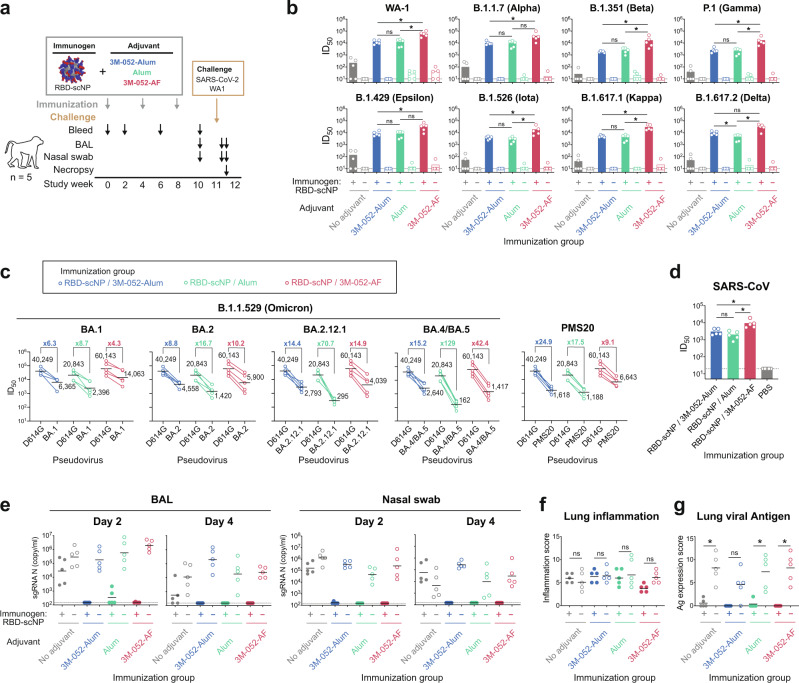
Neutralizing antibodies and in vivo protection elicited by RBD-scNP vaccine formulated with three different adjuvants. **a** Schematic of the vaccination and challenge study. Cynomolgus macaques (*n* = 5 per group) were immunized intramuscularly 3 times with 100 μg of RBD-scNP adjuvanted with 3M-052-Alum, Alum, 3M052-AF, or PBS control. Animals injected with adjuvant alone or PBS were set as control groups. Monkeys were then challenged with SARS-CoV-2 WA-1, subjected to blood, Bronchoalveolar lavage (BAL) and nasal swab collection, and necropsied for pathologic analysis. **b** Neutralization titers (ID_50_) of plasma antibodies against pseudovirus of SARS-CoV-2 variants in 293T-ACE2-TMPRSS2. Each dot indicates one monkey (*n* = 5 per group) and bars indicate geometric mean values of each group. Adjusted *p*-values: ns, not significant, *p < 0.05, two-sided Wilcoxon rank sum exact test. **c** Serum antibody neutralization against pseudoviruses of the SARS-CoV-2 Omicron (BA.1, BA.2, BA.2.12.1, BA.4/BA.5) variants, and SARS-CoV-2 PMS20 variant in 293T-ACE2 cells. The geometric mean titers and the fold reduction compared to D614G are shown. **d** Serum antibody neutralization against pseudoviruses of SARS-CoV in 293T-ACE2 cells. Each dot indicates one monkey (*n* = 5 per group) and bars indicate geometric mean values of each group. Adjusted p-values: ns, not significant, *p < 0.05, two-sided Wilcoxon rank sum exact test. **e** SARS-CoV-2 N gene sgRNA in BAL and nasal swab samples collected on day 2 and 4 post-challenge. Each dot indicates one monkey (*n* = 5 per group) and bars indicate geometric mean values of each group. Dashed line indicates limit of the detection. **f**, **g** Histopathological analysis. Lung sections from each animal were scored for lung inflammation by haematoxylin and eosin (H&E) staining (**f**), and for SARS-CoV-2 nucleocapsid antigen (Ag) expression by immunohistochemistry (IHC) staining (**g**). Each dot indicates one monkey (*n* = 5 per group) and bars indicate geometric mean values of each group. Adjusted *p*-values: ns not significant, *p < 0.05, two-sided Wilcoxon rank sum exact test. Source data are provided as a Source Data file.

To optimize adjuvant formulations for the RBD-scNP vaccine^10^, we studied the immunogenicity of the RBD-scNP immunogen with the TLR7/8 agonist 3M-052-AF, with aluminum hydroxide (Alum) alone, or with 3M-052-AF adsorbed to Alum (3M-052-Alum). Control groups included NHPs immunized with immunogen alone (RBD-scNP without adjuvant), adjuvant alone (3M-052-AF, Alum, or 3M-052-Alum without immunogen), or PBS alone (Fig. 1a). After three immunizations, RBD-scNP alone without adjuvant induced minimal binding antibodies to SARS-CoV-2 and other CoV spike antigens, whereas higher titers of binding antibodies were induced by RBD-scNP formulated with each adjuvant (Supplementary Fig. 1a). While all three adjuvant formulations were robustly immunogenic, RBD-scNP adjuvanted with 3M-052-AF induced the highest plasma antibodies that could block spike-binding to DH1047, which is a Group 7 RBD cross-neutralizing antibody^12–15^ (*p* < 0.05; Wilcoxon rank sum exact test; Supplementary Fig. 1b, c). Mucosal antibody levels tended to be comparable for macaques who received RBD-scNP formulated with 3M-052-AF or 3M-052+Alum, with lower titers seen when Alum was used (Supplementary Fig. 1d, e).

To determine if RBD-scNP-induced antibodies could neutralize SARS-CoV-2 variants, the neutralization capacity against ancestral and variant pseudoviruses was assessed for macaque plasma two weeks after the 3^rd^ RBD-scNP immunization (Fig. 1a). While the RBD-scNP alone group showed minimal neutralizing antibody titers, the RBD-scNP + 3M-052-AF group had remarkably high pseudovirus neutralizing antibody titers against SARS-CoV-2 WA-1 strain with geometric mean titer (GMT) ID_50_ of 59,497. The GMT ID_50_ of RBD-scNP + 3M-052-Alum and RBD-scNP + Alum groups against WA-1 were 12,267 and 12,610, respectively (Fig. 1b). Moreover, RBD-scNPs + 3M-052-AF immunized animals exhibited the highest magnitudes of neutralizing antibodies against each variant tested (Fig. 1b and Supplementary Fig. 1f), as well as for a constructed variant PMS20 with anticipated SARS-CoV-2 escape mutations^11^ (Supplementary Table 1). Although reduced ID_50_ titers were observed to different extents for the variants, because of the high initial neutralizing titers for the D614G strain, with 3M052-AF, the resulting ID_50_ titers ranged from 1417 to 14,063 for all the Omicron sublineage variants, including BA.1, BA.2, BA.2.12.1, and BA.4/BA.5 (Fig. 1c and Supplementary Table 1). PMS20 neutralization was comparable to that of Omicron BA.2 with a titer of 6643 (Fig. 1c). Thus, while the protective ID_50_ neutralization titer is not known yet for Omicron or its variants, in each case in our study the ID_50_ neutralization titer was far higher than the ~1:200 ID_50_ that is anticipated to the correlate of protection from SARS-CoV-2 WA-1 strain transmission^16–19^.

In addition, cross-neutralizing antibodies against SARS-CoV were detected in RBD-scNP-immunized animals. The 3M-052-AF adjuvated group showed significantly higher SARS-CoV neutralization titers (ID_50_ GMT = 9449) than the 3M-052-Alum adjuvated group (ID_50_ GMT = 3039) or the Alum adjuvated group (ID_50_ GMT = 2033) (Fig. 1d). Thus, the 3M-052-AF adjuvant induced higher neutralization titers against SARS-CoV-2 variants and SARS-CoV than either 3M-052-Alum or Alum alone.

### In vivo protection induced by adjuvanted RBD-scNP

To compare in vivo protection of RBD-scNP in the upper (nasal swab) and lower (bronchoalveolar lavage, BAL) respiratory tracts with different adjuvant formulations, cynomolgus macaques were challenged with the WA-1 strain of SARS-CoV-2 three weeks after the third immunization (Fig. 1a). Compared to unimmunized monkeys, the adjuvant alone groups exhibited similar or higher copies of envelope (E) and nucleocapsid (N) gene subgenomic RNA (sgRNA), and the RBD-scNP immunogen alone reduced sgRNA copies by only ~1-2 logs (Fig. 1e and Supplementary Fig. 1g). Immunization with either RBD-scNP adjuvanted with 3M-052-Alum or 3M-052-AF conferred robust protection against SARS-CoV-2 infection in both the upper and lower airways. Macaques in these groups had under-detection-limit or near-baseline levels of sgRNA N and E in both lower and upper respiratory tracts, demonstrating that adjuvant was required for eliciting protection from SARS-CoV-2 challenge. RBD-scNP + Alum immunized macaques showed positive E or N gene sgRNA in 1 of 5 and 2 of 5 macaques, respectively, in BAL samples collected on day 2 post-challenge. By day 4 post-challenge, all RBD-scNP adjuvanted groups showed no detectable sgRNA (Fig. 1e and Supplementary Fig. 1g).

Animals were necropsied 4 days after challenge for histopathologic analysis to determine SARS-CoV-2-associated lung inflammation. Histologic analysis of lung tissue by haematoxylin and eosin (H&E) staining showed that RBD-scNP + adjuvant and adjuvant only groups had similar inflammation scores (Fig. 1f). Immunohistochemistry (IHC) staining of the lung tissues exhibited high SARS-CoV-2 nucleocapsid antigen expression in the unimmunized and adjuvant alone groups. In contrast, 1 of 5 of the RBD-scNP + Alum immunized animals and 2 of 5 of the immunogen alone immunized animals had low level nucleocapsid antigen expression, and no lung viral nucleocapsid antigen was detected by IHC in the RBD-scNP plus 3M-052-Alum or RBD-scNP plus 3M-052-AF immunized animals (Fig. 1g). Therefore, the three adjuvanted vaccines conferred comparable protection against viral replication by day 4 post-challenge, with 3M-052-AF and 3M-052-Alum adjuvants providing optimal viral suppression.

### RBD-scNP, NTD-scNP, and S2P-scNP vaccines induced both neutralizing and ADCC-mediating antibodies

**Fig. 2:**
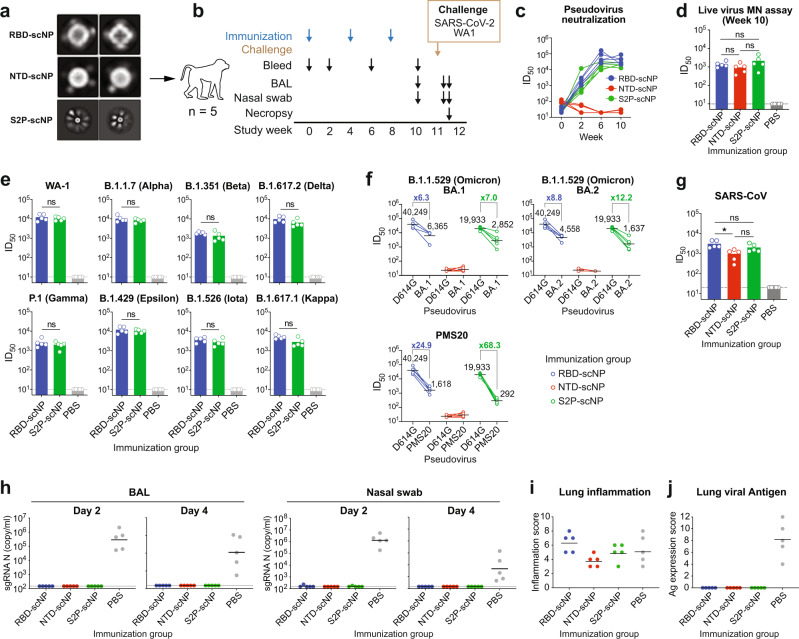
Neutralizing antibodies and in vivo protection induced by RBD-scNP, NTD-scNP and S2P-scNP vaccines. **a** Negative-stain electron microscopy 2D class averaging of RBD-scNP, NTD-scNP, and S2P-scNP. The 2D class averaging of 14,300 RBD-scNP particles, 10,800 NTD-scNP particles or 1034 S2P-scNP particles were generated using RELION. The size of each box: RBD-scNP and NTD-scNP, 257 Å; S2P-scNP, 1029 Å. **b** Schematic of the three-dose regimen. Cynomolgus macaques (*n* = 5 per group) were immunized 3 times with RBD-scNP, NTD-scNP, or S2P-scNP adjuvanted with 3M-052-Alum. Monkeys were then challenged with SARS-CoV-2 WA-1, sampled for blood, BAL and nasal swabs, and necropsied for pathologic analysis. **c** Neutralization titers of plasma antibodies (week 0, 2, 6 and 10) against pseudotyped SARS-CoV-2 D614G strain in 293T/ACE2.MF cells. **d** Neutralization titers of plasma antibodies (week 10) against live SARS-CoV-2 WA-1 virus in Vero-E6 cells in microneutralization (MN) assay. Each dot indicates one monkey (*n* = 5 per group) and bars indicate geometric mean values of each group. Adjusted *p*-values: ns not significant, *p < 0.05, two-sided Wilcoxon rank sum exact test. **e** Neutralization titers of plasma antibodies (*n* = 5 per group) against pseudoviruses of the SARS-CoV-2 variants in 293T-ACE2-TMPRSS2 cells. Each dot indicates one monkey (*n* = 5 per group) and bars indicate geometric mean values of each group. Adjusted *p*-values: ns, not significant, *p < 0.05, Two-sided Wilcoxon rank sum exact test. **f**, **g** Serum antibody neutralization against pseudoviruses of (**f**) the SARS-CoV-2 Omicron BA.1 and BA.2 variants, the PMS20 variant, and (**g**) SARS-CoV in 293T-ACE2 cells. Each dot indicates one monkey (*n* = 5 per group) and bars indicate geometric mean values of each group. For SARS-CoV-2, the geometric mean titers and the fold reduction compared to D614G are shown. Adjusted p-values: ns, not significant, *p < 0.05, Two-sided Wilcoxon rank sum exact test. **h** SARS-CoV-2 N gene sgRNA in BAL and nasal swab samples collected on day 2 and 4 post-challenge. **i**, **j** Histopathological analysis. Scores of lung inflammation were determined by H&E staining (**i**) and SARS-CoV-2 nucleocapsid antigen expression were determined by IHC staining (**j**). Source data are provided as a Source Data file.

While most of neutralizing antibodies target the RBD, neutralizing antibodies can target other sites on spike^20^. Thus, we also generated NTD- and stabilized spike S2P-scNPs and compared the antibody response elicited by these nanoparticle vaccines to that induced by RBD-scNPs (Fig. 2a, b and Supplementary Fig. 2a–c). Cynomolgus macaques were given three monthly immunizations with one of the scNPs formulated with 3M-052-Alum. After three immunizations, spike binding, ACE2-blocking, and neutralizing antibody-blocking antibodies were observed in all three groups (Supplementary Fig. 2d–f). In the RBD-scNP and S2P-scNP immunized animals, neutralizing antibodies against SARS-CoV-2 D614G pseudovirus or against live SARS-CoV-2 WA-1 virus in a microneutralization (MN) assay were detected after the first dose and were boosted after the second and third dose at week 6 and 10 (Fig. 2c, d). The NTD-scNP-induced sera contained IgGs that blocked NTD neutralizing antibody DH1050.1 binding (Supplementary Fig. 2e). However, NTD-scNP-sera post 2^nd^ and 3^rd^ immunization failed to neutralize the SARS-CoV-2 pseudovirus (Fig. 2c) but neutralized live SARS-CoV-2 WA-1 virus at similar titers (GMT ID_50_ = 2189) as RBD-scNP-sera or S2P-scNP-sera (Fig. 2d). The phenomenon of NTD antibodies neutralizing SARS-CoV-2 in live virus assays but not in pseudovirus assays has been previously reported^12,21,22^. Importantly, S2P-scNP induced comparable plasma neutralizing antibody titers compared to RBD-scNP against SARS-CoV-2 WA-1 strain and eight variants tested (*p* > 0.05; Wilcoxon rank sum exact test; Fig. 2e and Supplementary Fig. 2g). However, RBD-scNP induced higher neutralizing antibodies than S2P-scNP against pseudoviruses of Omicron BA.1 (GMT ID_50_ = 6365 for RBD-scNP versus 2852 for S2P-scNP), BA.2 (GMT ID_50_ = 4558 for RBD-scNP versus 1637 for S2P-scNP), and PMS20 (GMT ID_50_ = 1618 for RBD-scNP versus 292 for S2P-scNP) (Fig. 2f). Moreover, all three scNP vaccines induced cross-neutralizing antibodies against SARS-CoV (Fig. 2g). Notably, unlike SARS-CoV-2, the SARS-CoV pseudovirus could be neutralized by NTD antibodies. Nevertheless, the RBD-scNP-induced SARS-CoV neutralizing antibody titers were significantly higher than NTD-scNP-induced antibody titers.

To examine other antibody functions, we examined plasma antibody binding to cell surface-expressed SARS-CoV-2 spike and antibody-dependent cellular cytotoxicity (ADCC). Plasma antibodies induced by three doses of RBD-scNP, NTD-scNP and S2P-scNP vaccination bound to SARS-CoV-2 spike on the surface of transfected cells (Supplementary Fig. 2h**)**. In a CD107a degranulation-based ADCC assay (Supplementary Fig. 2i), plasma antibodies from all three scNP groups mediated CD107a degranulation of human NK cells in the presence of both SARS-CoV-2 spike-transfected cells and SARS-CoV-2-infected cells (Supplementary Fig. 2j). Thus, all three scNP vaccines induced antibodies that both neutralized SARS-CoV-2 and mediated ADCC.

### RBD-scNP, NTD-scNP, and S2P-scNP vaccines protect macaques against SARS-CoV-2 WA-1 challenge

To determine NTD-scNP and S2P-scNP immunization conferred protection against SARS-CoV-2, we challenged the immunized macaques with SARS-CoV-2 WA-1 strain via the intratracheal and intranasal routes one month after the 3^rd^ vaccination. All macaques that received RBD-scNP, NTD-scNP or S2P-scNP were fully protected, showing undetectable or near-detection-limit E or N gene sgRNA (Fig. 2h and Supplementary Fig. 2k). IHC staining of the lung tissues demonstrated high SARS-CoV-2 nucleocapsid protein expression in the control animals, whereas no viral antigen was detected in any of the scNP-immunized animals (Fig. 2i, j). The upper and lower airway sgRNA and lung immunohistochemistry data demonstrated that three doses of NTD-scNP or S2P-scNP immunization provided the same in vivo protection as RBD-scNP immunization.

### RBD-scNP, NTD-scNP and S2P-scNP as boosts for mRNA-LNP vaccine

**Fig. 3:**
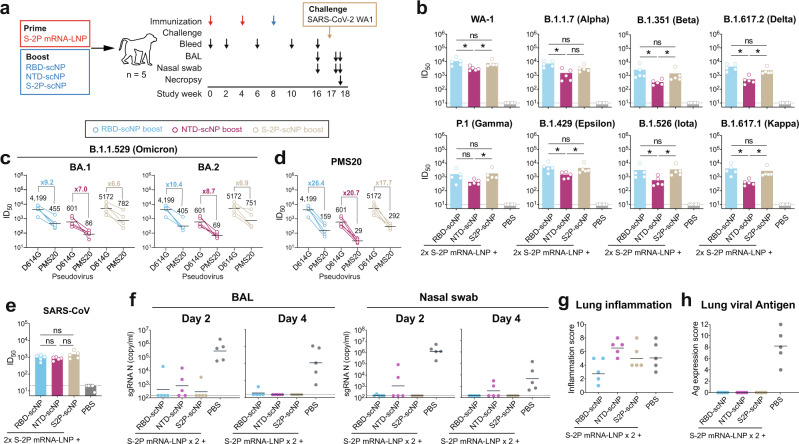
RBD-scNP, NTD-scNP and S2P-scNP vaccines as a heterologous boost for the S2P mRNA-LNP vaccine. **a** Schematic of the heterologous prime-boost regimen. Cynomolgus macaques (*n* = 5 per group) were immunized 2 times with S2P mRNA-LNP, and boosted with adjuvanted RBD-scNP, NTD-scNP, or S2P-scNP vaccine. Monkeys were then challenged with SARS-CoV-2 WA-1, sampleded for blood, BAL and nasal swabs, and necropsied for pathologic analysis. **b** Neutralization titers of plasma antibodies against pseudoviruses of SARS-CoV-2 variants in 293T-ACE2-TMPRSS2 cells. Each dot indicates one monkey (*n* = 5 per group) and bars indicate geometric mean values of each group. Adjusted *p*-values: ns not significant, *p < 0.05, Two-sided Wilcoxon rank sum exact test. Serum antibody neutralization titers against pseudoviruses of (**c**) the SARS-CoV-2 Omicron BA.1 and BA.2 variants, (**d**) the SARS-CoV-2 PMS20 variant, and (**e**) SARS-CoV in 293T-ACE2 cells. Each dot indicates one monkey (*n* = 5 per group) and bars indicate geometric mean values of each group. For SARS-CoV-2, the geometric mean titers and the fold reduction compared to D614G are shown. Adjusted *p*-values: ns not significant, *p < 0.05, Two-sided Wilcoxon rank sum exact test. **f** SARS-CoV-2 N gene sgRNA in BAL and nasal swab samples collected on day 2 and 4 post-challenge. Histopathological analysis. Scores of lung inflammation were determined by H&E staining (**g**) and SARS-CoV-2 nucleocapsid antigen expression were determined by IHC staining (**h**). Source data are provided as a Source Data file.

We next assessed the efficacy of the RBD-scNP, NTD-scNP and S2P-scNP as boosts in macaques that received two priming doses of mRNA vaccine. Cynomolgus macaques (*n* = 5) were immunized twice with 50 μg of S-2P-encoding, nucleoside-modified mRNA encapsulated in lipid nanoparticles (S-2P mRNA-LNP), which phenocopies the Pfizer/BioNTech and the Moderna COVID-19 vaccines. Subsequently, macaques were boosted with RBD-, NTD- or S2P-scNPs (Fig. 3a). Plasma antibody binding patterns were similar among the three groups until animals received the scNP boosting (Supplementary Fig. 3a). Plasma antibodies targeting to ACE2-binding site and neutralizing epitopes were detected after the scNP boosting, with cross-reactive antibodies to the pan-Sarbecovirus antibody DH1047-binding site^12–15^ being highest after RBD-scNP or S2P-NP boosting (Supplementary Fig. 3b, c). For mucosal antibody responses, BAL and nasal wash mucosal ACE2-blocking and DH1047-blocking activities tended to be low in magnitude in macaques primed with a Spike mRNA-LNP vaccine and boosted with RBD-scNP or S2P-scNP (Supplementary Fig. 3d, e).

Serum neutralizing titers against the WA-1 strain pseudovirus were similar in the RBD-scNP-boosted group (GMT ID_50_ = 10,912.1) and S2P-scNP-boosted group (GMT ID_50_ = 7799.9) (Fig. 3b), while the NTD-scNP-boosted group showed significantly lower titers (GMT ID_50_ = 3229.8; *p* = 0.027, exact Wilcoxon test). The same ranking of vaccine groups was also observed for neutralization of other variants (Fig. 3b). In addition, in the RBD-scNP- and S2P-scNP-boosted groups, reduced ID_50_ titers were mostly seen for the Beta and Gamma variants, whereas in the NTD-scNP-boosted group, Alpha, Beta, Gamma, Delta, Iota and Kappa variants all showed >5-fold reduction of ID_50_ titers (Supplementary Fig. 3f), indicating the NTD-scNP was an inferior boost of the S-2P mRNA-LNP vaccine compared to RBD- or S2P-scNPs. Two doses of S2P mRNA-LNP immunization and one dose of RBD-scNP, NTD-scNP, or S2P-scNP-boost induced neutralizing antibodies to D614G with GMT ID_50_ of 4,199, 601, and 5172, respectively, which dropped 6.6- to 26.4-folds when testing for BA.1, BA.2 and PMS20 pseudoviruses (Fig. 3c, d). The RBD-scNP-boost, NTD-scNP-boost, and S2P-scNP-boost all induced cross-neutralizing antibodies against SARS-CoV, with ID_50_ GMTs of 946, 824, and 1502, respectively (Fig. 3e). Thus, the mRNA-LNP prime/RBD-scNP one-month boost showed limited boosting capacity for neutralizing antibodies against Omicron, PMS20 or SARS-CoV, demonstrating a longer boosting interval will be needed as is used for boosting the current COVID-19 vaccines^23,24^.

We also measured T cell responses to pooled SARS-CoV-2 Spike peptides in macaques immunized with mRNA-LNP prime and scNP boost. Intracellular cytokine staining assays after the last immunization showed Spike-specific T helper 1 (TH1) responses in CD4 T cells, with >0.1% of IL-2+ CD4 T cells and TNF-α+ CD4 T cells in the RBD-scNP-boosted and S2P-scNP-boosted animals (Supplementary Fig. 3g). In addition, comparing to three doses of RBD-scNP vaccination, mRNA-LNP prime and RBD-scNP boost induced significantly more IL-2+ CD4 T cells and TNF-α+ CD4 T cells (Supplementary Fig. 3g).

This data demonstrated that the scNP boost only induced marginal T cell responses, which did not alter the TH1-biased CD4 T cell responses induced by mRNA-LNP vaccine as previously reported^16^.

Macaques that received mRNA-LNP prime and scNP boost at one-month post-mRNA-LNP primes were challenged with SARS-CoV-2 WA-1 strain after boosting. Four of five RBD-scNP-boosted monkeys and four of five of the S2P-scNP-boosted monkeys were completely protected from SARS-CoV-2 infection, showing no detectable E or N gene sgRNA in either BAL or nasal swab samples (Fig. 3f and Supplementary Fig. 3h). However, in the NTD-scNP boost group, N gene sgRNA was detected in BAL from three of five animals and in nasal swab samples from two of five animals (Fig. 3f). Macaques that received mRNA-LNP prime and RBD-scNP boost had the lowest degree of lung inflammation (Fig. 3g). In addition, no viral antigen was observed in lung tissues from either of the immunized groups as indicated by IHC staining for SARS-CoV-2 N protein (Fig. 3h). Thus, boosting selectively with the NTD provided less effective protection than boosting with S2P- or RBD-scNPs.

### Two doses of RBD-scNP immunization protected macaques from SARS-CoV-2 WA-1, Beta and Delta challenge

**Fig. 4:**
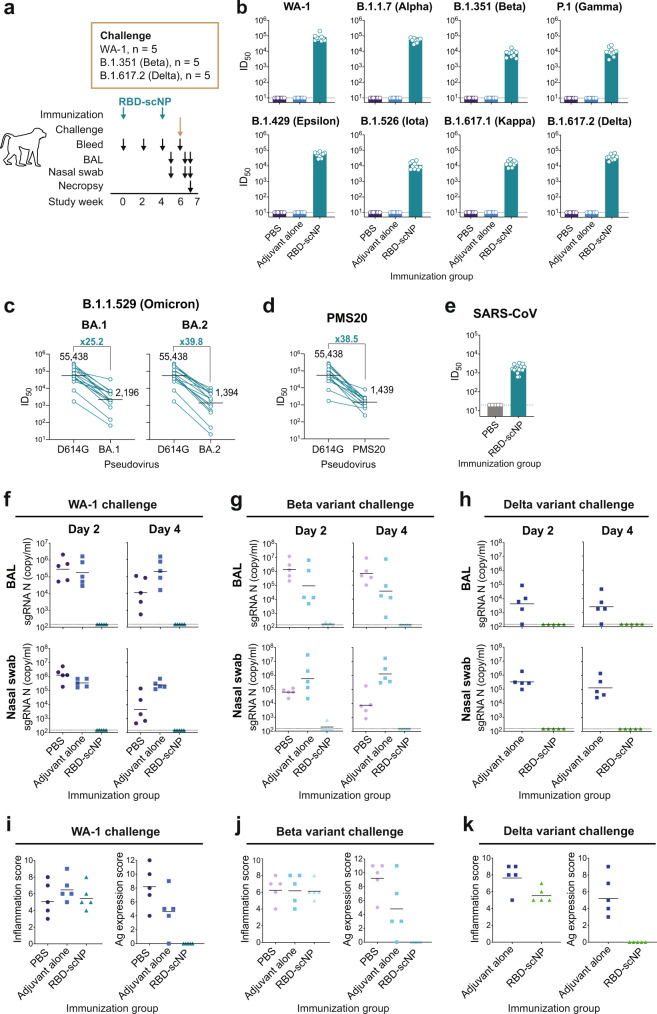
Two doses of RBD-scNP vaccination protected non-human primates from challenges of SARS-CoV-2 variants. **a** Schematic of the vaccination and challenge studies. Cynomolgus macaques were immunized twice with RBD-scNP adjuvanted with 3M-052-Alum, and challenged with SARS-CoV-2 WA-1 strain (*n* = 5) or B.1.351 (Beta; *n* = 5) or B.1.617.2 (Delta; *n* = 5), collected for blood, BAL and nasal swab samples, and necropsied for pathologic analysis. **b** Neutralization titers of plasma antibodies against pseudoviruses of SARS-CoV-2 variants in 293T-ACE2-TMPRSS2 cells. Each dot indicates one monkey (*n* = 10 per group) and bars indicate geometric mean values of each group. Adjusted *p*-values: ns not significant, *p < 0.05, Two-sided Wilcoxon rank sum exact test. Neutralization titers of serum antibodies against pseudoviruses of (**c**) the SARS-CoV-2 Omicron BA.1 and BA.2 variants, (**d**) the SARS-CoV-2 PMS20 variant, and (**e**) SARS-CoV in 293T-ACE2 cells. Each dot indicates one monkey (*n* = 15 per group) and bars indicate geometric mean values of each group. Adjusted p-values: ns, not significant, *p < 0.05, Two-sided Wilcoxon rank sum exact test. For SARS-CoV-2 variants, the geometric mean ID_50_ titers and the fold reduction compared to D614G are shown. SARS-CoV-2 sgRNA levels for nucleocapsid (N) gene in BAL and nasal swab samples collected on day 2 and 4 after (**f**) SARS-CoV-2 WA-1, (**g**) Beta variant or (**h**) Delta variant challenge. Dashed line indicates limit of the detection (LOD). Histopathological analysis of the SARS-CoV-2 (**i**) WA-1, (**j**) Beta variant or (**k**) Delta variant challenged monkeys. Scores of lung inflammation determined by H&E staining and SARS-CoV-2 nucleocapsid Ag expression determined by IHC staining. Source data are provided as a Source Data file.

Next, we determined if two doses of RBD-scNP vaccination could protect NHPs from challenge by SARS-CoV-2 WA-1, Beta or Delta variants. We immunized cynomolgus macaques with two doses of RBD-scNP vaccine, PBS, or adjuvant alone (Fig. 4a). RBD-scNP immunization elicited robust binding antibodies to SARS-CoV-2 and other CoV spike antigens (Supplementary Fig. 4a), as well as blocking antibodies to the ACE2-, DH1041- (a class 1 RBD non-cross-reactive neutralizing antibody), and DH0147-binding sites (Supplementary Fig. 4b, c). Two doses of RBD-scNP immunization induced neutralizing antibodies against all tested variants (Fig. 4b), although reduced neutralizing titers were observed for the Beta, Gamma, Kappa, and Iota variants compared to WA-1 (Supplementary Fig. 4d). In addition, serum antibodies induced by two doses of RBD-scNP immunization exhibited modest neutralization against BA.1 (GMT ID_50_ = 2196), BA.2 (GMT ID_50_ = 1394), and PMS20 (GMT ID_50_ = 1439), with 25.2-, 39.8-, and 38.5-fold drops compared to D614G neutralization titers (Fig. 4c, d). Lastly, SARS-CoV pseudovirus was neutralized by RBD-scNP-induced serum antibodies (GMT ID_50_ = 1752; Fig. 4e). Thus, although RBD-scNP induced neutralizing antibodies against SARS-CoV-2 variants and SARS-CoV after two immunizations, it showed far more fold-reduction in Omicron and PMS20 neutralization titers compared with three doses of RBD-scNP immunizations (Fig. 2f), demonstrating that a third boost was effective in enhancing neutralization breadth to the Omicron variants.

Two weeks after the second vaccination, macaques (*n* = 5 per group) were challenged with SARS-CoV-2 WA-1, SARS-CoV-2 B.1.351 (Beta) variant, or SARS-CoV-2 B.1.617.2 (Delta) variant (Fig. 4a). In the PBS or adjuvant alone group, high copies of E and N gene sgRNA were detected in both BAL and nasal swab samples collected on day 2 and 4 post-challenge (Fig. 4f–h and Supplementary Fig. 4e–g). In contrast, all animals in the RBD-scNP group were completely protected from WA-1 infection, as indicated by no detectable sgRNA in either BAL or nasal swab (Fig. 4f). After the SARS-CoV-2 Beta variant challenge, nasal N gene sgRNA was detected in only 1 of 5 of the RBD-scNP immunized monkeys (Fig. 4g), whereas after the SARS-CoV-2 Delta variant challenge, all animals that received two doses of RBD-scNP immunization showed no detectable sgRNA in BAL or nasal swab samples (Fig. 4h). Lung tissue H&E staining revealed no significant difference in inflammation scores between groups (Fig. 4i–k). However, IHC staining showed the presence of SARS-CoV-2 nucleocapsid antigen in the lungs of macaques administered PBS or adjuvant alone, but not in the lungs of RBD-scNP-immunized monkeys (Fig. 4i–k). Thus, two doses of RBD-scNP immunization protected against viral replication of WA-1, Beta variant, or Delta variant in both lower and upper airways.

### Two doses of RBD-scNP immunization induced protective responses in mice against SARS-CoV-2 Beta variant and other sarbecoviruses

**Fig. 5:**
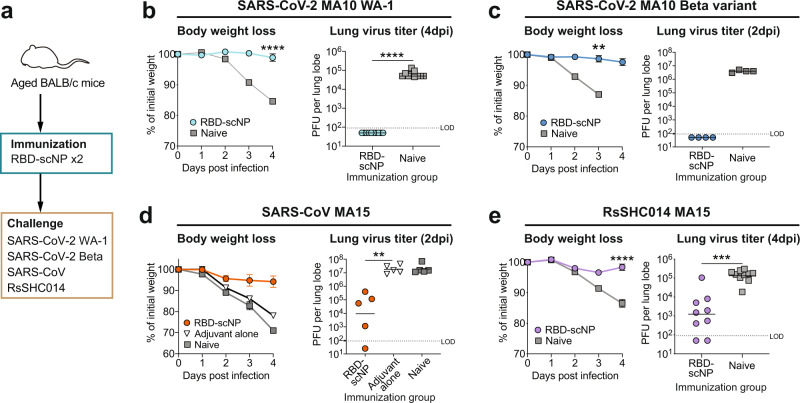
Two doses of RBD-scNP vaccination protected mice from challenges of SARS-CoV-2 variants and other betacoronaviruses. **a** Schematic of the mouse challenge studies. 11-month-old female BALB/c mice (*n* = 10 per group) were immunized intramuscularly twice with adjuvanted RBD-scNP and challenged with SARS-CoV-2 mouse-adapted 10 (MA10) WA-1, SARS-CoV-2 MA10 Beta variant, SARS-CoV-1 mouse-adapted 15 (MA15), or Bat coronavirus (CoV) RsSHC014 MA15. GLA-SE was used as adjuvant in the SARS-CoV challenge study, and 3M-052-Alum was used in the other challenge studies. **b** Weight loss (*n* = 10 per group) and lung virus titers (*n* = 10 per group) at 4 days post-infection (dpi) of the SARS-CoV-2 MA10 WA-1 challenged mice. **c** Weight loss (*n* = 5 per group) and lung virus titers (*n* = 4 per group) at 2 dpi of the SARS-CoV-2 MA10 Beta variant challenged mice. **d** Weight loss (*n* = 10 per group) and lung virus titers (*n* = 5 per group) at 2 dpi of the SARS-CoV-1 MA15 challenged mice. **e** Weight loss (*n* = 10 per group) and lung virus titers (*n* = 10 per group) at 4 dpi of the Bat CoV RsSHC014 MA15 challenged mice. For weight curves, data are presented as mean values ±SEM. For lung virus titers, each dot indicates one mouse and bars indicate geometric mean values of each group. *P*-values: ns not significant, *p < 0.05, **p < 0.01, ***p < 0.001, ****p < 0.0001, Two-sided Wilcoxon rank sum exact test. Source data are provided as a Source Data file.

To define the protective efficacy of the RBD-scNP vaccination against different sarbecoviruses after two immunizations, we immunized mice with two doses of RBD-scNPs, challenged the mice with mouse-adapted SARS-CoV-2 WA-1, SARS-CoV-2 Beta variant, SARS-CoV, or bat CoV RsSHC014 (Fig. 5a). Two doses of RBD-scNP immunization induced high titer binding antibodies to Spike proteins of SARS-CoV-2, SARS-CoV, and RsSHC014 (Supplementary Fig. 5a–c). In the SARS-CoV-2 WA-1 challenge study, RBD-scNP protected mice from weight loss through 4 days post infection (dpi) and protected from viral replication in the lungs (Fig. 5b). Similar protection from weight loss and lung viral replication were observed in the SARS-CoV-2 Beta variant challenged mice (Fig. 5c); moreover, by 4 days post infection mortality was observed in the unimmunized mice group but no RBD-scNP-immunized mice died. Mice immunized with RBD-scNP were also protected from weight loss induced by SARS-CoV infection and showed ~3-log lower average PFU titer in lungs compared to adjuvant alone and unimmunized groups (Fig. 5d). Lastly, RBD-scNP immunization conferred protection against the bat sarbecovirus RsSHC014 challenge-induced weight loss and resulted in ~2-log lower average PFU titer than naïve mice (Fig. 5e). Thus, two doses of RBD-scNP immunization elicited protective immune responses against SARS-CoV-2 Beta variant and other sarbecoviruses in mouse models.

## Discussion

In this study, the SARS-CoV-2 WA-1 RBD induced neutralizing antibodies to conserved epitopes among Beta, Delta and Omicron variants, despite the up to 17 RBD amino acid changes for the Omicron BA.2.12.1 or BA.4/BA.5 variant relative to the WA-1 strain. Given the neutralization titers that have protected macaques in challenge studies, we speculate the RBD-scNP vaccine would protect against Omicron variants challenge with similar efficacy as shown here for the Beta and Delta variants, as pseudovirus neutralizing antibody titers of approximately 50 were required for protection in monkeys^19^. Thus, one advantage of the scNP platform is that very high titers of neutralizing antibodies are induced such that even with a high degree of variant escape, the remaining variant neutralization titers are sufficiently high to protect against transmission.

In our previous study^10^ and here, we have shown that three immunizations with RBD-scNP can protect against SARS-CoV-2 WA1 challenge. The purpose of the 2-dose RBD-scNP protection study was to test if the third dose RBD-scNP booster was necessary for macaque protection. Although 2-dose RBD-scNP protected monkeys from challenge, ID_50_ titers against Omicron could be boosted by the third dose, with GMT ID50 fold-change against BA.2 being 39.8-fold after 2-dose immunization and 8.8-fold after 3-dose immunization, demonstrating the necessity for the third dose of vaccination for robust neutralizing antibody titers.

While SARS-CoV-2 continues to mutate during the ongoing pandemic, there are conserved RBD neutralizing epitopes among the SARS-CoV-2 variants. This result is supported by studies with the SARS-CoV-2 virus PMS20 where 20 naturally occurring mutations were introduced into the spike protein, but the resultant virus was still sensitive to human vaccine-induced polyclonal antibodies^11^ and in our study, scNP-induced serum antibodies in our study. For present and future coverage of SARS-CoV-2 variants, it will be critical to induce a polyclonal response that targets conserved sites on the RBD. Monoclonal antibodies such as S2x259, S2K146^25^, DH1047^12,15^ and S309^26^ have defined key conserved RBD sites upon which vaccines can be designed. It is important to study the induction of broadly neutralizing antibodies by heterologous prime-boost regimens, as a large proportion of the global population have been vaccinated with the WA-1 spike. In the present study, mRNA prime-scNP boost-induced neutralizing antibody titers against the Omicron variants were similar to that elicited by a third dose of an mRNA vaccine^27,28^. Future efforts should be focused on developing the RBD-scNP vaccine as a booster for the currently vaccinated population, to optimize the induction of pan-sarbecovirus RBD neutralizing antibodies in preparation for the future SARS-CoV-2 variants or another CoV outbreak.

Adjuvants play essential roles in vaccine formulation to elicit strong protective immune responses^29^ and Alum is used in many currently approved vaccines^30^. Thus, it was encouraging to see that the RBD-NP vaccine was protective in NHPs when adsorbed to Alum. Compared to Alum, 3M-052-AF demonstrated superior capacities to elicit neutralizing antibodies against SARS-CoV-2 variants when formulated with SARS-CoV-2 RBD trimer in macaques^31^. In addition, 3M-052-adjuvanted gp140 Env vaccine induced high-titer neutralizing antibodies against tier 1A HIV-1 pseudovirus in rhesus macaques^32^. 3M-052-Alum is being used in clinical trials evaluating HIV-1 vaccines (NCT04915768 and NCT04177355). Here we found that 3M-052-AF-adjuvanted RBD-scNPs induced not only superior systemic and mucosal binding antibody responses, but also higher titers of neutralizing antibodies than 3M-052-Alum-adjuvanted vaccine, demonstrating that 3M-052-AF in the absence of Alum is an optimal adjuvant for scNP. One explanation for this difference could be antagonism between Th1-based immune pathways induced by 3M-052^33^ and Th2-based pathways induced by Alum^34^. Another potential explanation is that physicochemical considerations such as particle size or adsorption interactions between Alum and the RBD-scNP antigen and/or 3M-052-AF are impacting vaccine biodistribution, presentation, or cellular processing, thus affecting downstream immune responses. Such interactions are antigen-dependent^35^, highlighting the importance of optimizing adjuvant formulation for each antigen type^30,36^. Coronavirus vaccines formulated with Alum have been reported to be associated with enhanced lung inflammation, particularly with killed vaccines^37,38^. However, it is important to note that no enhancement of lung inflammation or virus replication was seen with RBD-scNP/Alum formulations. The RBD-scNP + 3M-052-AF group exhibited the highest neutralizing antibody titers and was the only group showing reduced severity of lung inflammation.

While nanoparticle vaccines could effectively prevent viral replication in both lower and upper respiratory tracts, they failed to prevent the immune response to the instillation of the challenge virus directly into the trachea. That lung inflammation was present suggested an intense immune response to the challenge virus. While differential lung inflammation is suggested, no statistically significant differences was observed except for the RBD-scNP + 3M-052-AF group. That the 3M-052-AF group exhibited the highest neutralizing antibody titers and showed reduced severity of lung inflammation demonstrated that 3M-052-AF was the optimal adjuvant formulation tested for the RBD-NP vaccine.

While the RBD subunit has been shown to protect against SARS-CoV-2 challenge in animal models^10,39–42^, the NTD is also an immunodominant region for neutralizing antibodies^12,21,22,43–45^. However, NTD is the site of multiple mutations and NTD antibody neutralization is, in general, less potent than RBD antibodies. Here, in this study, NTD-scNP-induced serum neutralizing antibodies were detected using a live SARS-CoV-2 WA-1 strain D614G virus but not using pseudovirus. The inconsistent neutralization activities of NTD antibodies in different neutralization assays have been previously observed^12,21^. In addition, the NTD-scNP immunization likely induced not only neutralizing, but also non-neutralizing NTD antibodies that can confer partial protection from SARS-CoV-2 challenge in macaques^12^. Serum from the NTD-scNP group did have ADCC activity, suggesting that non-neutralizing Fc receptor-mediated antibody activities could have been involved in protection. In this regard, we previously found that a non-neutralizing NTD antibody DH1052 provided partial protection from WA-1 challenge after infusion into mice and non-human primates^12^. Therefore, the complete protection conferred by scNP vaccination could be a result of both neutralizing and non-neutralizing Fc receptor-mediated antibody activities. Moreover, we found that boosting with RBD-scNP or S2P-scNP after S2P mRNA-LNPs priming afforded complete protection for monkeys after WA-1 challenge, while NTD-scNP boosting of S2P mRNA-LNPs priming led to incomplete protection. The mechanism of this latter finding is currently under investigation.

Our study has several limitations. First, our study did not evaluate the durability of vaccine-induced immune responses and protection against SARS-CoV-2 variants. Second, we were unable to set up longer time intervals between the second and the third booster vaccination, to mimic 4–6 month boosting interval in humans. Third, transmission studies in mouse models would be important to evaluate the capacity of nanoparticle vaccines preventing transmission of SARS-CoV-2 and other sarbecoviruses. Lastly, we only were able to challenge the animals with WA-1 strain, the Beta variant or the Delta variant. Future in vivo protection studies will be required upon availability of robust viral stocks of other SARS-CoV-2 variants such as the Omicron variants BA.2, BA.2.12.1 and BA.4/BA.5 become available.

Thus, our study demonstrates that scNP platform vaccines with different SARS-CoV-2 spike subunits confer potent protection in NHPs against WA-1, Beta and Delta variants, and induce high titers of neutralizing antibodies to SARS-CoV-2 variants. These findings have important implications for protection from virus escape from neutralizing antibody responses and for development of the next generation of COVID-19 vaccines.

## Methods

### Animals and immunizations

The study protocol and all veterinarian procedures were approved by the Bioqual IACUC per a memorandum of understanding with the Duke IACUC, and were performed based on standard operating procedures. Macaques studied were housed and maintained in an Association for Assessment and Accreditation of Laboratory Animal Care-accredited institution in accordance with the principles of the National Institutes of Health. All studies were carried out in strict accordance with the recommendations in the Guide for the Care and Use of Laboratory Animals of the National Institutes of Health in BIOQUAL (Rockville, MD). BIOQUAL is fully accredited by AAALAC and through OLAW, Assurance Number A-3086. All physical procedures associated with this work were done under anesthesia to minimize pain and distress in accordance with the recommendations of the Weatherall report, “The use of non-human primates in research.” Teklad 5038 Primate Diet was provided once daily by animal size and weight. The diet was supplemented with fresh fruit and vegetables. Fresh water was given ad libitum. All monkeys were maintained in accordance with the Guide for the Care and Use of Laboratory Animals.

Cynomolgus macaques were on average 8–9 years old and ranged from 2.75 to 8 kg in body weight. Male and female macaques per group were balanced when availability permitted. Studies were performed unblinded. The immunogens were formulated with adjuvants and given intramuscularly in the right and left quadriceps. In the adjuvant study (Fig. 1 and Supplementary Fig. 1), cynomolgus macaques were immunized for twice with 100 μg of RBD-scNP or recombinant soluble RBD with 5 μg of 3M-052 aqueous formulation admixed with 500 μg of alum in PBS. In the fourth study, macaques were divided into 8 groups (*n* = 5 per group) as following: 1) control group: no immunization; 2) immunogen alone group: 100 μg of RBD-scNP; 3) RBD-scNP + 3M-052-Alum group: 100 μg of RBD-scNP + 5 μg of 3M-052 in aqueous formulation + 500 μg of Alum (i.e. aluminum ion); 4) 3M-052-Alum alone group: 5 μg of 3M-052 in aqueous formulation + 500 μg of Alum; 5) RBD-scNP + Alum group: 100 μg of RBD-scNP + 500 μg of Alum; 6) Alum alone group: 500 μg of Alum; 7) RBD-scNP + 3M-052-AF group: 100 μg of RBD-scNP + 5 μg of 3M-052 in aqueous formulation; 8) 3M-052-AF alone group: 5 μg of 3M-052 in aqueous formulation. In the RBD-, NTD-, and S2P-scNP study (Fig. 2 and Supplementary Fig. 2), cynomolgus macaques (*n* = 5) were immunized for three times with 100 μg of RBD-scNP, NTD-scNP and S2P-scNP adjuvanted with 5 μg of 3M-052 aqueous formulation admixed with 500 μg of alum in PBS. In the prime-boost study (Fig. 3 and Supplementary Fig. 3), cynomolgus macaques (*n* = 5) were were immunized twice with 50 μg of S-2P mRNA-LNP (encoding the transmembrane spike protein stabilized with K986P and V987P mutations) and boosted once with 100 μg of RBD-scNP, NTD-scNP and S2P-scNP adjuvanted with 5 μg of 3M-052 aqueous formulation admixed with 500 μg of alum in PBS. In the last RBD-scNP 2× immunization study (Fig. 4 and Supplementary Fig. 4), cynomolgus macaques (*n* = 5) were immunized for two times with 100 μg of RBD-scNP adjuvanted with 5 μg of 3M-052 aqueous formulation admixed with 500 μg of alum in PBS.

### SARS-CoV-2 viral challenge

For SARS-CoV-2 challenge, 10^5^ plaque-forming units (PFU) of SARS-CoV-2 virus Isolate USA-WA1/2020, or 2 × 10^5^ PFUs of SARS-CoV-2 Beta or Delta variants, were diluted in 4 mL and were given by 1 mL intranasally and 3 mL intratracheally on Day 0. Biospecimens, including nasal swabs, BAL, plasma, and serum samples, were collected before immunization, after every immunization, before challenge, 2 days post-challenge and 4 days post-challenge. Animals were necropsied on Day 4 post-challenge, and lungs were collected for histopathology and immunohistochemistry (IHC) analysis.

### Recombinant protein production

The coronavirus ectodomain proteins were produced and purified as previously described^10,12,46,47^. S-2P was stabilized by the introduction of 2 prolines at amino acid positions 986 and 987. Plasmids encoding SARS-CoV-2 and other coronavirus S-2P (Genscript) were transiently transfected in FreeStyle 293-F cells (Thermo Fisher) using Turbo293 (SpeedBiosystems) or 293Fectin (ThermoFisher). All cells were tested monthly for mycoplasma. The constructs contained an HRV 3C-cleavable C-terminal twinStrepTagII-8×His tag. On day 6, cell-free culture supernatant was generated by centrifugation of the culture and filtering through a 0.8-μm filter. Protein was purified from filtered cell culture supernatants by StrepTactin resin (IBA) and by size-exclusion chromatography using Superdex 200 (RBD and NTD) or Superose 6 (S-2P and ferritin) column (GE Healthcare) in 10 mM Tris pH = 8500 mM NaCl. ACE2-Fc was expressed by transient transfection of Freestyle 293-F cells. ACE2-Fc was purified from cell culture supernatant by HiTrap protein A column chromatography and Superdex200 size-exclusion chromatography in 10 mM Tris pH8,150 mM NaCl. SARS-CoV-2 RBD and NTD were produced as previously described^10,47^.

RBD-scNP, NTD-scNP, and S2P-scNP were produced by conjugating SARS-CoV-2 RBD to *H. pylori* ferritin nanoparticles using Sortase A as previously described^10^. Briefly, SARS-CoV-2 Wuhan strain RBD, NTD or S-2P (with a C-terminal foldon trimerization motif) was expressed with a sortase A donor sequence LPETGG encoded at its C terminus. C-terminal to the sortase A donor sequence was an HRV-3C cleavage site, 8×His tag and a twin StrepTagII (IBA). The proteins were expressed in Freestyle 293-F cells and purified by StrepTactin affinity chromatography and Superdex 200 or Superose 6 size-exclusion chromatography. *Helicobacter pylori* ferritin particles were expressed with a pentaglycine sortase A acceptor sequence encoded at its N terminus of each subunit. For affinity purification of ferritin particles, 6×His tags were appended C-terminal to a HRV3C cleavage site. Ferritin particles with a sortase A N-terminal tag were buffer exchanged into 50 mM Tris, 150 mM NaCl, 5 mM CaCl_2_, pH 7.5. Then 180 μM SARS-CoV-2 RBD was mixed with 120 μM of ferritin subunits and incubated with 100 μM of sortase A overnight at room temperature. Following incubation, conjugated particles were isolated from free ferritin or free RBD/NTD/S-2P by size-exclusion chromatography using a Superose 6 16/60 column. Western blots of reduced RBD, NTD, or Spike ectodomain scNPs were performed to determine the approximate amount of coronavirus protein within the 100 μg immunogen dose. Mouse polyclonal antisera against ferritin was used to blot for ferritin that was either unconjugated or conjugated to coronavirus proteins. Bio-Rad ImageDoc system was used to quantify the pixels of the ferritin conjugate band and unconjugated ferritin band. The percentage of conjugated ferritin was determined as the pixels of the conjugated ferritin divided by the sum of the pixels for the ferritin conjugate band and unconjugated ferritin band multiplied by 100 percent. The average percentages of conjugated ferritin were 80%, 70%, and 94.1%, for RBD, NTD, or Spike ectodomain respectively. We approximate the micrograms of RBD, NTD, or Spike ectodomain per 100 micrograms of immunogen to be 86, 55, and 51 micrograms respectively.

### Negative-stain electron microscopy

The RBD, NTD, or S-2P nanoparticle protein at about 1–5 mg ml−1 concentration that had been flash-frozen and stored at −80 °C was thawed in an aluminum block at 37 °C for 5 min; then 1–4 μl of RBD, NTD, or S-2P nanoparticle was diluted to a final concentration of 0.1 mg ml−1 into room-temperature buffer containing 150 mM NaCl, 20 mM HEPES pH 7.4, 5% glycerol and 7.5 mM glutaraldehyde. After 5 min of cross-linking, excess glutaraldehyde was quenched by adding sufficient 1 M Tris pH 7.4 stock to give a final concentration of 75 mM Tris and incubated for 5 min. For negative stain, carbon-coated grids (EMS, CF300-cu-UL) were glow-discharged for 20 s at 15 mA, after which a 5-μl drop of quenched sample was incubated on the grid for 10–15 s, blotted and then stained with 2% uranyl formate. After air drying, grids were imaged with a Philips EM420 electron microscope operated at 120 kV, at 82,000× magnification and images captured with a 2k × 2k CCD camera at a pixel size of 4.02 Å.

### Processing of negative-stain images

The RELION 3.0 program was used for all negative-stain image processing. Images were imported, CTF-corrected with CTFFIND and particles were picked using a nanoparticle template from previous 2D class averages of nanoparticles alone. Extracted particle stacks were subjected to 2 or 3 rounds of 2D class averaging and selection to discard junk particles and background picks.

### mRNA-LNP vaccine production

The S-2P mRNA was designed based on the SARS-CoV-2 spike (S) protein sequence (Wuhan-Hu-1) and encoded the full-length S with K986P and V987P amino acid substitutions. Production of the mRNA was performed as described earlier^48,49^. Briefly, the codon-optimized S-2P gene was synthesized (Genscript) and cloned into an mRNA production plasmid. A T7-driven in vitro transcription reaction (Megascript, Ambion) using linearized plasmid template was performed to generate mRNA with 101 nucleotide long poly(A) tail. Capping of the mRNA was performed in concert with transcription through addition of a trinucleotide cap1 analog, CleanCap (TriLink) and m1Ψ-5’-triphosphate (TriLink) was incorporated into the reaction instead of UTP. Cellulose-based purification of S-2P mRNA was performed as described^50^. The S-2P mRNA was then tested on an agarose gel before storing at −20 °C. The cellulose-purified m1Ψ-containing S-2P mRNA was encapsulated in LNPs using a self-assembly process as previously described wherein an ethanolic lipid mixture of ionizable cationic lipid, phosphatidylcholine, cholesterol and polyethylene glycol-lipid was rapidly mixed with an aqueous solution containing mRNA at acidic pH^51^. The RNA-loaded particles were characterized and subsequently stored at 80 °C at a concentration of 1 mg/ml.

### Antibody binding ELISA

For binding ELISA, 384-well ELISA plates were coated with 2 μg/mL of antigens in 0.1 M sodium bicarbonate overnight at 4 °C. Plates were washed with PBS +0.05% Tween 20 and blocked with blocked with assay diluent (PBS containing 4% (w/v) whey protein, 15% Normal Goat Serum, 0.5% Tween-20, and 0.05% Sodium Azide) at room temperature for 1 h. Plasma or mucosal fluid were serially diluted threefold in superblock starting at a 1:30 dilution. Nasal fluid was started from neat, whereas BAL fluid was concentrated ten-fold. To concentrate BAL, individual BAL aliquots from the same macaque and same time point were pooled in 3-kDa MWCO ultrafiltration tubes (Sartorious, catalog # VS2091). Pooled BAL was concentrated by centrifugation at 3200 × *g* for 30 min or until volume was reduced by a factor of 10. The pool was then aliquoted and frozen at −80 °C until its use in an assay. Serially diluted samples were added and incubated for 1 h, followed by washing with PBS-0.1% Tween 20. HRP-conjugated goat anti-human IgG secondary Ab (SouthernBiotech, catalog# 2040-05) was diluted to 1:10,000 and incubated at room temperature for 1 h. These plates were washed four times and developed with tetramethylbenzidine substrate (SureBlue Reserve- KPL). The reaction was stopped with 1 M HCl, and optical density at 450 nm (OD_450_) was determined.

### ACE2 and neutralizing antibody blocking assay

ELISA plates were coated as stated above with 2 μg/mL recombinant ACE-2 protein or neutralizing antibodies, then washed and blocked with 3% BSA in 1x PBS. While assay plates blocked, plasma or mucosal samples were diluted as stated above, only in 1% BSA with 0.05% Tween-20. In a separate dilution plate spike-2P protein was mixed with the antibodies at a final concentration equal to the EC50 at which spike binds to ACE-2 protein. The mixture was incubated at room temperature for 1 h. Blocked assay plates were then washed and the antibody-spike mixture was added to the assay plates for a period of 1 h at room temperature. Plates were washed and a polyclonal rabbit serum against the same spike protein (nCoV-1 nCoV-2P.293F) was added for 1 h, washed and detected with goat anti rabbit-HRP (Abcam catalog # ab97080) followed by TMB substrate. The extent to which antibodies were able to block the binding spike protein to ACE-2 or neutralizing antibodies was determined by comparing the OD of antibody samples at 450 nm to the OD of samples containing spike protein only with no antibody. The following formula was used to calculate percent blocking: blocking % = (100 − (OD sample/OD of spike only)*100).

### Pseudotyped SARS-CoV-2 neutralization assay

Neutralization of SARS-CoV-2 Spike-pseudotyped virus was performed by adopting an infection assay described previously^52^ with lentiviral vectors and infection in 293T/ACE2.MF (the cell line was kindly provided by Drs. Mike Farzan and Huihui Mu at Scripps). Cells were maintained in DMEM containing 10% FBS and 50 µg/ml gentamicin. An expression plasmid encoding codon-optimized full-length spike of the Wuhan-1 strain (VRC7480), was provided by Drs. Barney Graham and Kizzmekia Corbett at the Vaccine Research Center, National Institutes of Health (USA). Mutations were introduced into VRC7480 either by site-directed mutagenesis using the QuikChange Lightning Site-Directed Mutagenesis Kit from Agilent Technologies (Catalog # 210518), or were created by spike gene synthesized by GenScript using the spike sequence in VRC7480 as template. All mutations (D614G, Omicron BA.1, BA.2, BA.2.12.1, BA.4/BA.5 and PMS20) were confirmed by full-length spike gene sequencing by Sanger Sequencing, using Sequencher and SnapGene for sequence analyses. Pseudovirions were produced in HEK 293T/17 cells (ATCC cat. no. CRL-11268) by transfection using Fugene 6 (Promega, Catalog #E2692). Pseudovirions for 293T/ACE2 infection were produced by co-transfection with a lentiviral backbone (pCMV ΔR8.2) and firefly luciferase reporter gene (pHR' CMV Luc)^53^. Culture supernatants from transfections were clarified of cells by low-speed centrifugation and filtration (0.45 µm filter) and stored in 1 ml aliquots at −80 °C. A pre-titrated dose of virus was incubated with 8 serial 3-fold or 5-fold dilutions of mAbs in duplicate in a total volume of 150 µl for 1 h at 37 °C in 96-well flat-bottom poly-L-lysine-coated culture plates (Corning Biocoat). Cells were suspended using TrypLE express enzyme solution (Thermo Fisher Scientific) and immediately added to all wells (10,000 cells in 100 µL of growth medium per well). One set of 8 control wells received cells + virus (virus control) and another set of 8 wells received cells only (background control). After 66–72 h of incubation, medium was removed by gentle aspiration and 30 µL of Promega 1x lysis buffer was added to all wells. After a 10-min incubation at room temperature, 100 µl of Bright-Glo luciferase reagent was added to all wells. After 1–2 min, 110 µl of the cell lysate was transferred to a black/white plate (Perkin-Elmer). Luminescence was measured using a PerkinElmer Life Sciences, Model Victor2 luminometer. Neutralization titers are the serum dilution (ID_50_/ID_80_) at which relative luminescence units (RLU) were reduced by 50% and 80% compared to virus control wells after subtraction of background RLUs. Negative neutralization values are indicative of infection-enhancement. Maximum percent inhibition (MPI) is the reduction in RLU at the highest mAb concentration tested.

Another protocol was used to test plasma neutralization against pseudoviruses of SARS-CoV-2 WA-1 strain and variants. Human codon-optimized cDNA encoding SARS-CoV-2 spike glycoproteins of various strains were synthesized by GenScript and cloned into eukaryotic cell expression vector pcDNA 3.1 between the BamHI and XhoI sites. Pseudovirions were produced by co-transfection of Lenti‐X 293T cells with psPAX2(gag/pol), pTrip-luc lentiviral vector and pcDNA 3.1 SARS-CoV-2-spike-deltaC19, using Lipofectamine 3000. The supernatants were collected at 48 h after transfection and filtered through 0.45-μm membranes and titrated using HEK293T cells that express ACE2 and TMPRSS2 protein (293T-ACE2-TMPRSS2 cells). For the neutralization assay, 50 μl of SARS-CoV-2 spike pseudovirions were pre-incubated with an equal volume of medium containing serum at varying dilutions at room temperature for 1 h, then virus-antibody mixtures were added to 293T-ACE2-TMPRSS2 cells in a 96-well plate. After a 3-h incubation, the inoculum was replaced with fresh medium. Cells were lysed 24 h later, and luciferase activity was measured using luciferin. Controls included cell-only control, virus without any antibody control and positive control sera. Neutralization titers are the serum dilution (ID50 or ID80) at which relative luminescence units (RLU) were reduced by 50% or 80%, respectively, compared to virus control wells after subtraction of background RLUs.

### Live SARS-CoV-2 neutralization assays

The SARS-CoV-2 virus (Isolate USA-WA1/2020, NR-52281) was deposited by the Centers for Disease Control and Prevention and obtained through BEI Resources, NIAID, NIH. SARS-CoV-2 Micro-neutralization (MN) assays were adapted from a previous study^54^. In short, sera or purified Abs are diluted two-fold and incubated with 100 TCID50 virus for 1 h. These dilutions are used as the input material for a TCID50. Each batch of MN includes a known neutralizing control Ab (Clone D001; SINO, CAT# 40150-D001). Data are reported as the concentration at which 50% of input virus is neutralized. A known neutralizing control antibody is included in each batch run (Clone D001; SINO, CAT# 40150-D001). GraphPad Prism was used to determine ID_50_ values.

### Spike protein-expressing cell antibody binding assay

The cell antibody binding assay was performed as previously described (Pino et al., 2021). Briefly, target cells were derived by transfection with plasmids designed to express the SARS-CoV-2 D614 Spike protein with a c-terminus flag tag (kindly provided by Dr. Farzan, Addgene plasmid no. 156420 (Zhang et al., 2020)). Cells not transfected with any plasmid (mock transfected) were used as a negative control condition. After resuspension, washing and counting, 1 × 10^5^ Spike-transfected target cells were dispensed into 96-well V-bottom plates and incubated with six serial dilutions of macaque plasma starting at 1:50 dilution. Mock transfected cells were used as a negative infection control. After 30 min incubation at 37 °C, cells are washed twice with 250 μL/well of PBS, stained with vital dye (Live/Dead Far Red Dead Cell Stain, Invitrogen) to exclude nonviable cells from subsequent analysis, washed with Wash Buffer (1%FBS-PBS; WB), permeabilized with CytoFix/CytoPerm (BD Biosciences), and stained with 1.25 µg/mL anti-human IgG Fc-PE/Cy7 (Clone HP6017; Biolegend) and 5 µg/mL anti-flag-FITC (clone M2; Sigma Aldrich) in the dark for 20 min at room temperature. After three washes with Perm Wash (BD Biosciences), the cells were resuspended in 125 μL PBS-1% paraformaldehyde. Samples were acquired within 24 h using a BD Fortessa cytometer and a High Throughput Sampler (HTS, BD Biosciences). Data analysis was performed using FlowJo 10.8.0 software. A minimum of 50,000 total events were acquired for each analysis. Gates were set to include singlet, live, flag+ and IgG+ events. All final data represent specific binding, determined by subtraction of non-specific binding observed in assays performed with mock-transfected cells.

### Antibody-dependent NK cell degranulation assay

Cell-surface expression of CD107a was used as a marker for NK cell degranulation, a prerequisite process for ADCC (Ferrari et al., 2011), was performed as previously described (Pino et al., 2021). Briefly, target cells were either Vero E6 cells after a 2 day-infection with SARS-CoV-2 USA-WA1/2020 or 293T cells 2-days post transfection with a SARS-CoV-2 S protein (D614) expression plasmid. NK cells were purified from peripheral blood of a healthy human volunteer, from the External Quality Assurance Program Oversight Laboratory (EQAPOL)^55^, in compliance with Institutional Review Board protocols approved by Duke University Medical Center. NK cells were by negative selection (Miltenyi Biotech), and were incubated with target cells at a 1:1 ratio in the presence of diluted plasma or monoclonal antibodies, Brefeldin A (GolgiPlug, 1 μl/ml, BD Biosciences), monensin (GolgiStop, 4 μl/6 mL, BD Biosciences), and anti-CD107a-FITC (BD Biosciences, clone H4A3) in 96-well flat bottom plates for 6 h at 37 °C in a humidified 5% CO2 incubator. NK cells were then recovered and stained for viability prior to staining with CD56-PECy7 (BD Biosciences, clone NCAM16.2), CD16-PacBlue (BD Biosciences, clone 3G8), and CD69-BV785 (Biolegend, Clone FN50). Flow cytometry data analysis was performed using FlowJo software (v10.8.0). Data is reported as the % of CD107A+ live NK cells (gates included singlets, lymphocytes, aqua blue-, CD56+ and/or CD16+, CD107A+). All final data represent specific activity, determined by subtraction of non-specific activity observed in assays performed with mock-infected cells and in absence of antibodies.

### Intracellular cytokine staining (ICS) assay

Cryopreserved PBMC were thawed and rested 4 h at 37 °C in a 5% CO_2_ environment. PBMC were then incubated for 6 h in the presence of either RPMI containing 10% fetal bovine serum (unstimulated), Staphylococcus enterotoxin B (SEB) as positive control, or pool peptide spanning the entire SARS-CoV-2 spike protein. All cultures contained a protein transport inhibitor, monensin (Golgi Plug; Becton, Dickinson and Company), and 1 μg/ml of anti-CD49d (Becton, Dickinson and Company, Cat# 340976). Cultured cells were then stained with a cell viability marker and pre-titered quantities of antibodies against CD3/CD4/CD8/CD45RA/ICOS/CCR7/CXCR3/PD-1/CXCR5/CD69/CD154/IL-2/IFN-g/TNF-a/IL-4/IL-21/IL-13/IL-17A. Samples with at least 1,000 viable CD4+ or CD8+ T cells were included. Samples were analyzed on a LSR II instrument (Becton, Dickinson and Company, Franklin Lakes, NJ) using FlowJo v10.8.1 software.

### Viral RNA extraction and subgenomic mRNA quantification

SARS-CoV-2 E gene and N gene subgenomic mRNA (sgRNA) was measured by a one-step RT-qPCR adapted from previously described methods^56,57^. To generate standard curves, a SARS-CoV-2 E gene sgRNA sequence, including the 5’UTR leader sequence, transcriptional regulatory sequence (TRS), and the first 228 bp of E gene, was cloned into a pcDNA3.1 plasmid. For generating SARS-CoV-2 N gene sgRNA, the E gene was replaced with the first 227 bp of N gene. The recombinant pcDNA3.1 plasmid was linearized, transcribed using MEGAscript T7 Transcription Kit (ThermoFisher, catalog # AM1334), and purified with MEGAclear Transcription Clean-Up Kit (ThermoFisher, catalog # AM1908). The purified RNA products were quantified on Nanodrop, serial diluted, and aliquoted as E sgRNA or N sgRNA standards.

A QIAsymphony SP (Qiagen, Hilden, Germany) automated sample preparation platform along with a virus/pathogen DSP midi kit. RNA extracted from animal samples or standards were then measured in Taqman custom gene expression assays (ThermoFisher). For these assays we used TaqMan Fast Virus 1-Step Master Mix (ThermoFisher, catalog # 4444432) and custom primers/probes targeting the E gene sgRNA (forward primer: 5′ CGA TCT CTT GTA GAT CTG TTC TCE 3′; reverse primer: 5′ ATA TTG CAG CAG TAC GCA CAC A 3′; probe: 5′ FAM-ACA CTA GCC ATC CTT ACT GCG CTT CG-BHQ1 3′) or the N gene sgRNA (forward primer: 5′ CGA TCT CTT GTA GAT CTG TTC TC 3′; reverse primer: 5′ GGT GAA CCA AGA CGC AGT AT 3′; probe: 5′ FAM-TAA CCA GAA TGG AGA ACG CAG TGG G-BHQ1 3′). RT-qPCR reactions were carried out on CFX384 Touch Real-Time PCR System (Bio-Rad) using a program below: reverse transcription at 50 °C for 5 min, initial denaturation at 95 °C for 20 s, then 40 cycles of denaturation-annealing-extension at 95 °C for 15 s and 60 °C for 30 s. Standard curves were used to calculate E or N sgRNA in copies per ml; the limit of detections (LOD) for both E and N sgRNA assays were 12.5 copies per reaction or 150 copies per mL of BAL/nasal swab.

### Histopathology

Lung specimen from nonhuman primates were fixed in 10% neutral buffered formalin, processed, and blocked in paraffin for histological analysis. All samples were sectioned at 5 µm and stained with hematoxylin-eosin (H&E) for routine histopathology. Sections were examined under light microscopy using an Olympus BX51 microscope and photographs were taken using an Olympus DP73 camera. Samples were scored by a board-certified veterinary pathologist in a blinded manner. The representative images are to characterize the types and arrangement of inflammatory cells, while the scores show the relative severity of the tissue section.

### Immunohistochemistry (IHC)

Staining for SARS-CoV-2 antigen was achieved on the Bond RX automated system with the Polymer Define Detection System (Leica) used per manufacturer’s protocol. Tissue sections were dewaxed with Bond Dewaxing Solution (Leica) at 72 °C for 30 min then subsequently rehydrated with graded alcohol washes and 1× Immuno Wash (StatLab). Heat-induced epitope retrieval (HIER) was performed using Epitope Retrieval Solution 1 (Leica), heated to 100 °C for 20 min. A peroxide block (Leica) was applied for 5 min to quench endogenous peroxidase activity prior to applying the SARS-CoV-2 antibody (1:2000, GeneTex, GTX135357). Antibodies were diluted in Background Reducing Antibody Diluent (Agilent). The tissue was subsequently incubated with an anti-rabbit HRP polymer (Leica) and colorized with 3,3’-Diaminobenzidine (DAB) chromogen for 10 min. Slides were counterstained with hematoxylin.

### Mouse immunization and challenge

Eleven-month-old female BALB/c mice were purchased from Envigo (#047) and were used for the SARS-CoV, SARS-CoV-2 WA-1, SARS-CoV-2 B.1.351, and RsSHC014-CoV protection experiments. The study was carried out in accordance with the recommendations for care and use of animals by the Office of Laboratory Animal Welfare (OLAW), National Institutes of Health and the Institutional Animal Care and Use Committee (IACUC) of University of North Carolina (UNC permit no. A-3410-01). Animals were housed in groups of five and fed standard chow diets. Virus inoculations were performed under anesthesia and all efforts were made to minimize animal suffering. Mice were intramuscularly immunized with 10 μg RBD-scNP formulated with 3M-052-Alum or GLA-SE. For the SARS-CoV-2 WA-1 and RsSHC014 study, mice were immunized on week 0 and 2, and challenged on week 7. For the SARS-CoV-2 B.1.351 and SARS-CoV study, mice were immunized on week 0 and 4, and challenged on week 6. All mice were anesthetized and infected intranasally with 1 × 10^4^ PFU/ml of SARS-CoV MA15, 1 × 10^4^ PFU/ml of SARS-CoV-2 WA1- MA10 or B.1.351-MA10, 1 × 10^4^ PFU/ml RsSHC014, which have been described previously^15,45,58–60^. Mice were weighted daily and monitored for signs of clinical disease, and selected groups were subjected to daily whole-body plethysmography. For all mouse studies, groups of *n* = 10 mice were included per arm of the study. Lung viral titers and weight loss were measured from individual mice per group.

### Biocontainment and biosafety

Studies were approved by the UNC Institutional Biosafety Committee approved by animal and experimental protocols in the Baric laboratory. All work described here was performed with approved standard operating procedures for SARS-CoV-2 in a biosafety level 3 (BSL-3) facility conforming to requirements recommended in the Microbiological and Biomedical Laboratories, by the U.S. Department of Health and Human Service, the U.S. Public Health Service, and the U.S. Center for Disease Control and Prevention (CDC), and the National Institutes of Health (NIH).

### Statistics analysis

Data were plotted using Prism GraphPad 8.0. Two-sided Wilcoxon rank sum exact test was performed to compare differences between groups with *p*-value < 0.05 considered significant using SAS 9.4 (SAS Institute, Cary, NC). The Benjamini-Hochberg correction^61^ was used to adjust the *p*-values for multiple comparisons.

### Reporting summary

Further information on research design is available in the Nature Research Reporting Summary linked to this article.

## Supplementary information

Supplementary Material

Reporting Summary

## Data Availability

The data that support the findings of this study are available from the corresponding authors upon reasonable request. Source data are provided with this paper.

## Source data

Source Data
