# Supplementary material for: Breadth of SARS-CoV-2 neutralization and protection induced by a nanoparticle vaccine
Source: Nat Commun. 2022 Oct 23;13:6309. doi: 10.1038/s41467-022-33985-4 (PMC9588772; doi:10.1038/s41467-022-33985-4)
Supplement: Supplementary file 1 — Supplementary Material [file 41467_2022_33985_MOESM1_ESM.pdf]

Supplementary Table 1

	PMS20	BA.1	BA.2	BA.2.12.1	BA.4/BA.5
L18F	■				
T19I			■	■	■
Δ24-26			■	■	■
A27S			■	■	■
V47M	■				
A67V		■			
Δ69-70	■	■			■
D80A	■				
T95I		■			
G142D		■	■	■	■
Δ143-145		■			
Δ145	■				
Δ211		■			
L212I		■			
V213G			■	■	■
ins214EPE		■			
D215G	■				
Δ242-244	■				
W258R	■				
G339D		■	■	■	■
R346S	■				
S371L		■			
S371F			■	■	■
S373P		■	■	■	■
S375F		■	■	■	■
T376A			■	■	■
D405N			■	■	■
R408S			■	■	■
K417N	■	■	■	■	■
N440K	■	■	■	■	■
V445E	■				
G446S		■			
L452Q				■	
L452R					■
L455R	■				
A475V	■				
S477N		■	■	■	■
T478K		■	■	■	■
E484A		■	■	■	■
E484K	■				
F486V					■
Q493R		■	■	■	
G496S		■			
Q498R		■	■	■	■
N501Y	■	■	■	■	■
Y505H		■	■	■	■
T547K		■			
D614G	■	■	■	■	■
H655Y		■	■	■	■
N679K		■	■	■	■
P681H		■	■	■	■
A701V	■				
S704L				■	
N764K		■	■	■	■
P792H	■				
D796Y		■	■	■	■
N801D	■				
N856K		■			
Q954H		■	■	■	■
N969K		■	■	■	■
L981F		■			

Supplementary Table 1. The mutations in the S protein of the SARS-CoV-2 Omicron BA.1, BA.2, BA.2.12.1, BA.4/BA.5 sublineages, and the PMS20 variant. Residues within the RBD region are in red.

Supplementary Figure 1

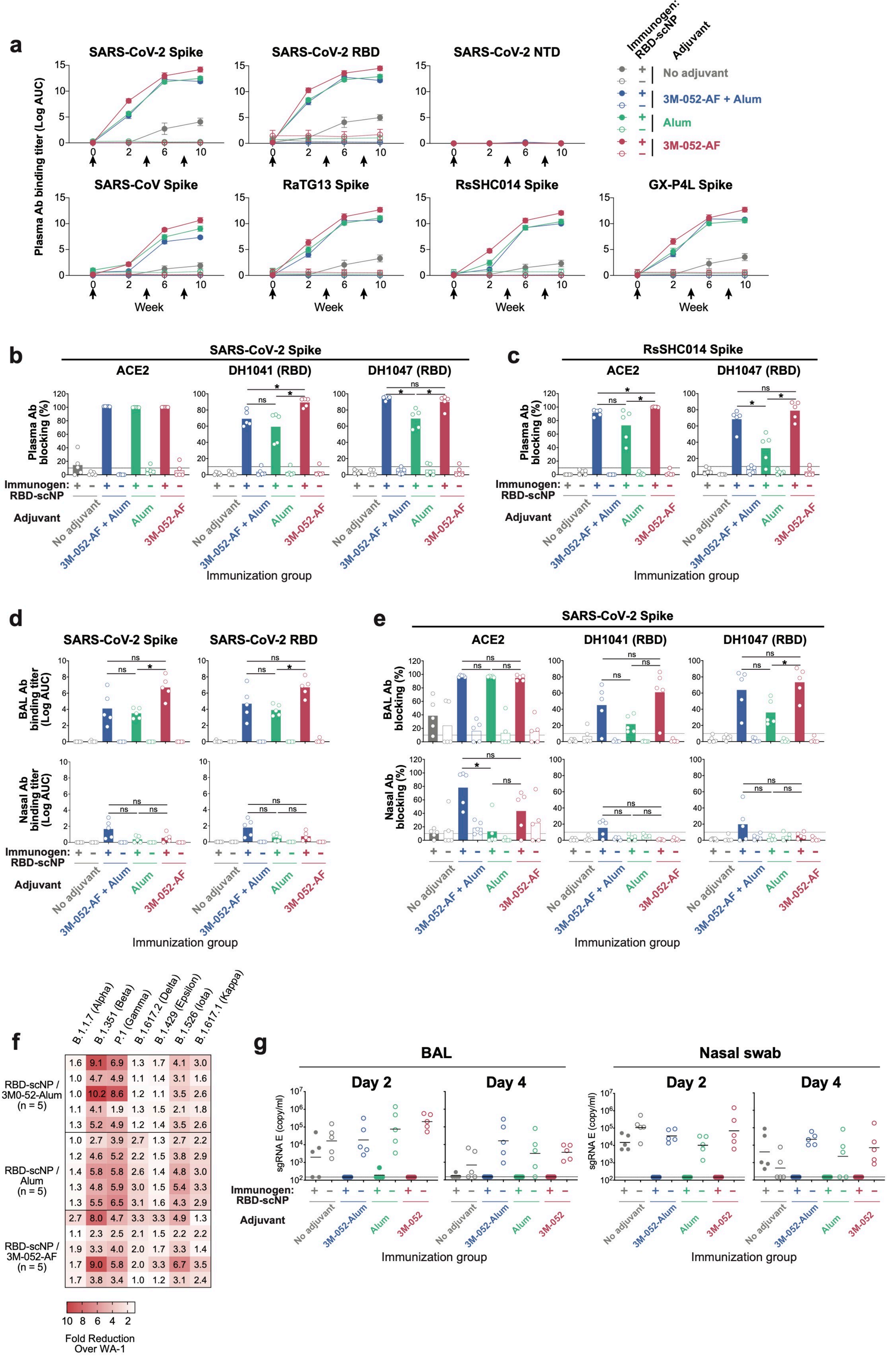

Supplementary Figure 1. RBD-scNP formulated with three different adjuvants induced humoral responses and protection against SARS-CoV-2 challenge in non-human primates. Related to Figure 1.
a. Plasma antibody binding titers to SARS-CoV-2 spike, RBD and NTD, as well as recombinant spike proteins of SARS-CoV, bat CoV RaTG13, RsSHC014, and pangolin CoV GX-P4L. ELISA binding titers are shown as mean ± SEM of log area-under-curve (AUC).
b-c. Plasma antibody (post-3rd immunization) blocking activity. ELISA was performed to test plasma antibodies blocking ACE2, human RBD neutralizing antibodies DH1041 and DH1047 binding to SARS-CoV-2 spike protein (b), or blocking ACE2 and DH1047 binding to RsSHC014 spike protein (c). Data are expressed as % blocking of ACE or the indicated antibody by 1:50 diluted plasma samples. Each dot indicates one monkey (n=5 per group) and bars indicate geometric mean values of each group. Adjusted p-values: ns, not significant, *$p<0.05$, Wilcoxon rank sum exact test.
d-e. Mucosal antibody binding and blocking activities after the 3rd immunization. ELISA was performed to test 10x concentrated BAL or unconcentrated nasal wash samples binding to SARS-CoV-2 spike and RBD (d), or blocking ACE2, DH1041 and DH1047 binding to SARS-CoV-2 spike protein (e). Binding titers are expressed as log AUC, and blocking activities are shown as %blocking of ACE or the indicated antibody. Each dot indicates one monkey (n=5 per group) and bars indicate geometric mean values of each group. Adjusted p-values: ns, not significant, *$p<0.05$, Wilcoxon rank sum exact test.
f. Reduction of ID_{50} titers against variants shown as fold reduction compared to the titers against WA-1.
g. SARS-CoV-2 E gene sgRNA in BAL and nasal swab samples from the WA-1 challenged monkeys. Dashed line indicates limit of the detection. Each dot indicates one monkey (n=5 per group) and bars indicate geometric mean values of each group.
Source data are provided as a Source Data file.

Supplementary Figure 2

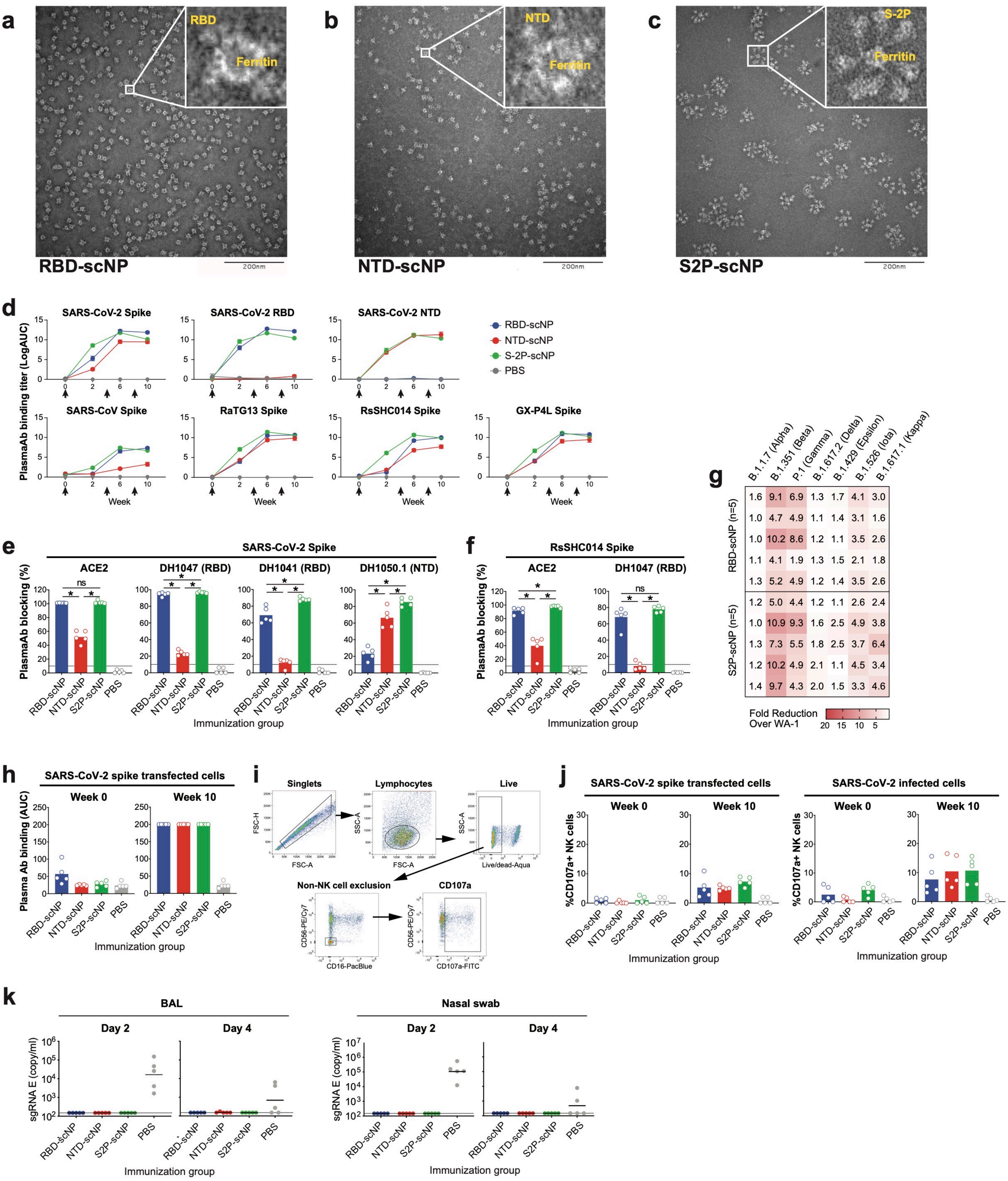

Supplementary Figure 2. Immune responses and *in vivo* protection elicited by RBD-scNP, NTD-scNP and S2P-scNP. Related to Figure 2.
a-c. Negative stain electron microscopy imaging of RBD-scNP (a), NTD-scNP (b), and S2P-scNP (c). The inset shows zoomed-in image of representative scNPs.
d. Plasma antibody binding titers to SARS-CoV-2 spike, RBD and NTD, as well as recombinant spike proteins of SARS-CoV, bat CoV RaTG13, RsSHC014, and pangolin CoV GX-P4L. ELISA binding titers are shown as mean ± SEM of log area-under-curve (AUC).
e-f. Plasma antibody (post-3rd immunization) blocking activity. ELISA was performed to test plasma antibodies blocking ACE2, human RBD neutralizing antibodies DH1041 and DH1047, human NTD neutralizing antibodies DH1050.1 binding to SARS-CoV-2 spike protein (E), or blocking ACE2 and DH1047 binding to RsSHC014 spike protein (F). Data are expressed as % blocking of ACE or the indicated antibody by 1:50 diluted plasma samples. Each dot indicates one monkey (n=5 per group) and bars indicate geometric mean values of each group. Adjusted p-values: ns, not significant, *p<0.05, Wilcoxon rank sum exact test.
g. Reduction of ID_{50} titers against variants were shown as fold reduction compared to the titers against WA-1
h. Pre-immunization (week 0) and pre-challenge (week 10, post-3rd immunization) plasma antibodies binding on SARS-CoV-2 spike-transfected 293T cells tested by cell surface staining. Each dot indicates one monkey (n=5 per group) and bars indicate geometric mean values of each group. Adjusted p-values: ns, not significant, *p<0.05, Wilcoxon rank sum exact test.
i. The gating strategy for the NK cell degranulation ADCC assay. Purified human NK cells were mixed with SARS-CoV-2 spike-transfected cells or SARS-CoV-2 infected cells in the presence of 1:50 diluted plasma samples. NK cell degranulation was detected based on CD107a expression.
j. RBD-scNP-, NTD-scNP- and S2P-scNP-induced antibodies mediated ADCC. The percentages of CD107a+ NK cells were shown when NK cells were assayed with plasma antibodies (week 0 and week 10) in SARS-CoV-2 spike transfected 293T cells or SARS-CoV-2 infected Vero E6 cells cells. Each dot indicates one monkey (n=5 per group) and bars indicate geometric mean values of each group. Adjusted p-values: ns, not significant, *p<0.05, Wilcoxon rank sum exact test.
k. SARS-CoV-2 E gene sgRNA in BAL and nasal swab samples from WA-1 challenged monkeys. Dashed line indicates limit of the detection. Each dot indicates one monkey (n=5 per group) and bars indicate geometric mean values of each group.
Source data are provided as a Source Data file.

Supplementary Figure 3

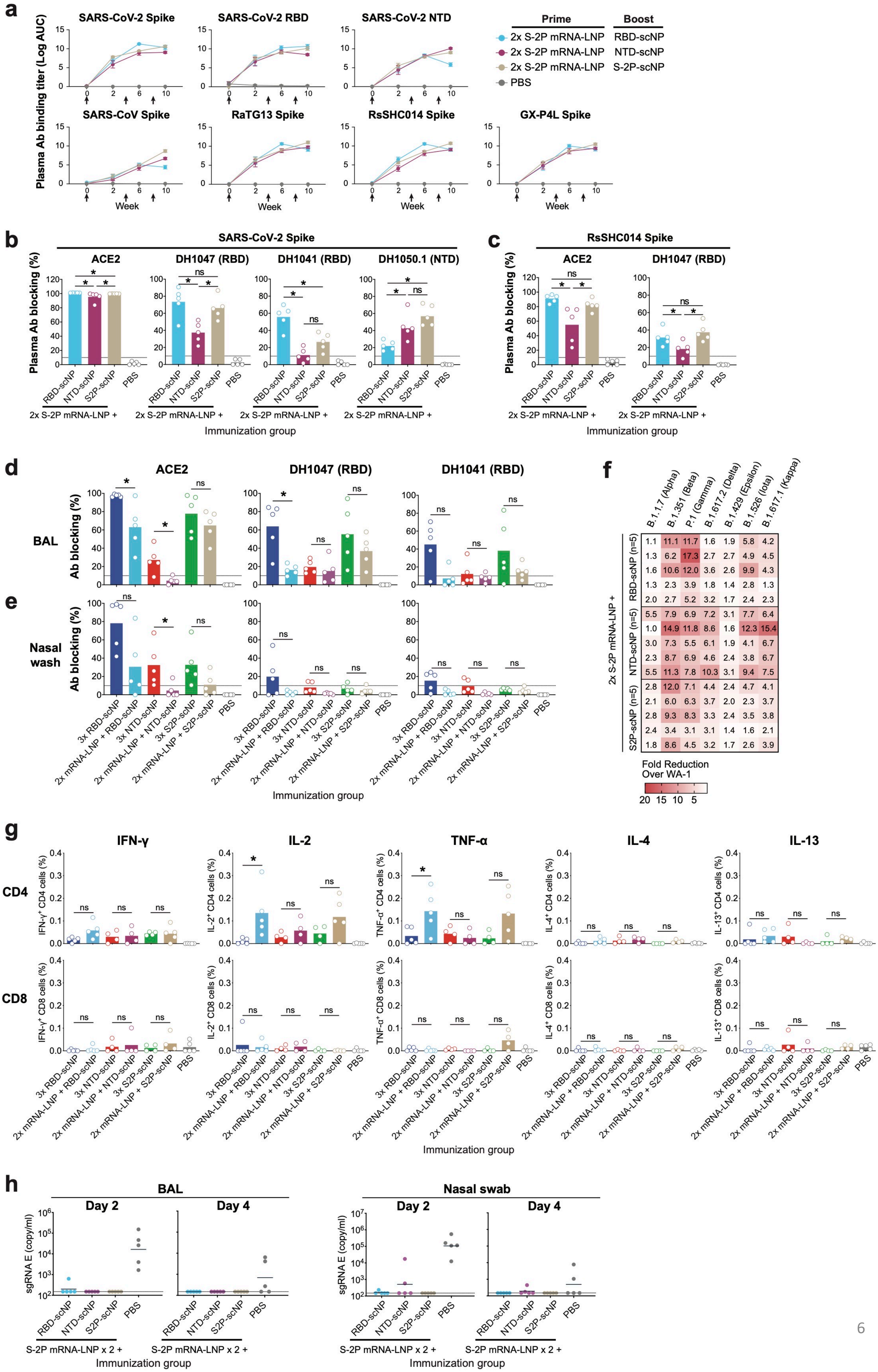

Supplementary Figure 3. scNP vaccines as a booster vaccination in macaques that received two doses of S-2P mRNA-LNP vaccine. Related to Figure 3.

a. Plasma antibody binding titers to SARS-CoV-2 spike, RBD and NTD, as well as recombinant spike proteins of SARS-CoV, bat CoV RaTG13, RsSHC014, and pangolin CoV GX-P4L. ELISA binding titers are shown as mean ± SEM of log area-under-curve (AUC).

b-c. Plasma antibody (post-2nd immunization) blocking activity. ELISA was performed to test plasma antibodies blocking ACE2, human RBD neutralizing antibodies DH1041 and DH1047, human NTD neutralizing antibodies DH1050.1 binding to SARS-CoV-2 spike protein (b), or blocking ACE2 and DH1047 binding to RsSHC014 spike protein (c). Data are expressed as % blocking of ACE or the indicated antibody by 1:50 diluted plasma samples. Each dot indicates one monkey (n=5 per group) and bars indicate geometric mean values of each group. Adjusted p-values: ns, not significant, *p<0.05, Wilcoxon rank sum exact test.

d-e. Comparison of mucosal antibody blocking activities induced by 3 doses of scNP vaccination or 2 doses of S2P mRNA-LNP + 1 dose of scNP vaccination. ELISA for 10x concentrated BAL samples (d) and neat nasal wash samples (e) blocking the binding of ACE2 or neutralizing antibody (DH1041 or DH1047) on SARS-CoV-2 spike were performed. Data are expressed as % blocking of ACE or the indicated antibody by mucosal samples. Each dot indicates one monkey (n=5 per group) and bars indicate geometric mean values of each group. Adjusted p-values: ns, not significant, *p<0.05, Wilcoxon rank sum exact test.

f. Reduction of ID_{50} titers against variants were shown as fold reduction compared to the titers against WA-1.

g. SARS-CoV-2-specific T cell responses induced by 3 doses of scNP vaccination or 2 doses of S2P mRNA-LNP + 1 dose of scNP vaccination. Intracellular staining (ICS) were performed in PBMCs collected after the last immunization. Th1 cytokine (IFN-γ, IL-2 and TNF-α)-secreting cells and Th2 cytokine (IL-4 and IL-13)-secreting cells in PBMC CD4+ and CD8+ T cells were measured. Each dot indicates one monkey (n=5 per group) and bars indicate geometric mean values of each group. Adjusted p-values: ns, not significant, *p<0.05, Wilcoxon rank sum exact test.

h. SARS-CoV-2 E gene sgRNA in BAL and nasal swab samples from WA-1 challenged monkeys. Each dot indicates one monkey (n=5 per group) and bars indicate geometric mean values of each group. Dashed line indicates limit of the detection.

Source data are provided as a Source Data file.

Supplementary Figure 4

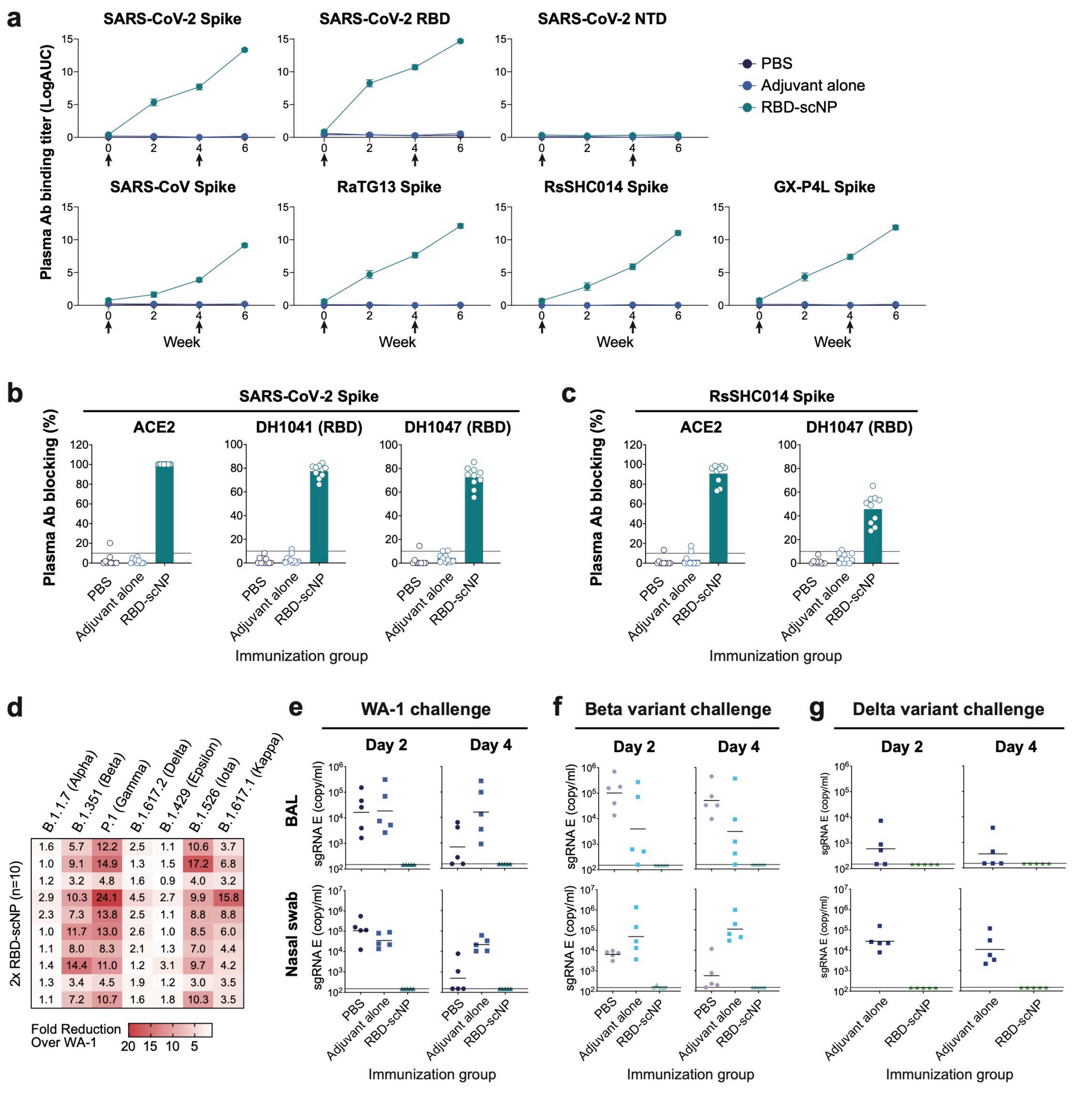

Supplementary Figure 4. Antibody responses and in vivo protection induced by two doses of RBD-scNP immunization. Related to Figure 4.

a. Plasma antibody binding titers to SARS-CoV-2 spike, RBD and NTD, as well as recombinant spike proteins of SARS-CoV, bat CoVs RaTG13, RsSHC014, and pangolin CoV GX-P4L. ELISA binding titers are shown as mean ± SEM of log area-under-curve (AUC).

b-c. Plasma antibody (post-2nd immunization) blocking activity. ELISA was performed to test plasma antibodies blocking ACE2, human RBD neutralizing antibodies DH1041 and DH1047 binding to SARS-CoV-2 spike protein (B), or blocking ACE2 and DH1047 binding to RsSHC014 spike protein (C). Data are expressed as % blocking of ACE or the indicated antibody by 1:50 diluted plasma samples. Each dot indicates one monkey (n=10 per group) and bars indicate geometric mean values of each group. Adjusted p-values: ns, not significant, *p<0.05, Wilcoxon rank sum exact test.

d. Fold reduction of plasma antibody ID_{50} titers against pseudoviruses of SARS-CoV-2 variants in 293T-ACE2-TMPRSS2 cells, compared to the titers against WA-1.

e-g. SARS-CoV-2 E gene sgRNA in BAL and nasal swab samples from the WA-1 (e), Beta variant (f), and Delta variant (g) challenged monkeys. Each dot indicates one monkey (n=5 per group) and bars indicate geometric mean values of each group. Dashed line indicates limit of the detection.

Source data are provided as a Source Data file.

Supplementary Figure 5

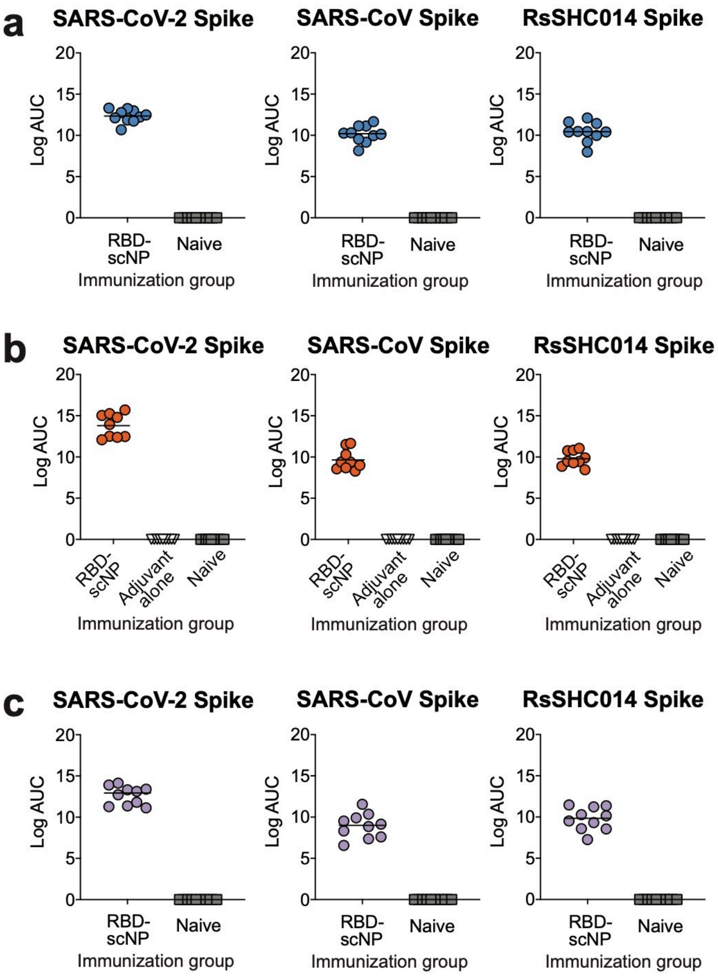

Supplementary Figure 5. Cross-reactive antibody responses induced by two doses of RBD-scNP immunization in mice. Plasma antibody binding titers (logAUC) to SARS-CoV-2 spike, SARS-CoV spike, and Bat CoV RsSHC014 spike were tested for the SARS-CoV-2 MA15 beta variant challenge study (a), SARS-CoV MA10 challenge study (b), and Bat CoV RsSHC014 MA15 challenge study (c) related to Figure 5. Each dot indicates one mouse (n=10 or 9 per group) and bars indicate geometric mean values of each group. Source data are provided as a Source Data file.
