# Supplementary material for: Breadth of SARS-CoV-2 neutralization and protection induced by a nanoparticle vaccine
Source: Nat Commun. 2022 Oct 23;13:6309. doi: 10.1038/s41467-022-33985-4 (PMC9588772; doi:10.1038/s41467-022-33985-4)
Supplement: Supplementary file 2 — Reporting Summary [file 41467_2022_33985_MOESM2_ESM.pdf]

nature portfolio

Corresponding author(s): Kevin Saunders

Last updated by author(s): Oct 1, 2022

Reporting Summary

Nature Portfolio wishes to improve the reproducibility of the work that we publish. This form provides structure for consistency and transparency in reporting. For further information on Nature Portfolio policies, see our Editorial Policies and the Editorial Policy Checklist.

Statistics

For all statistical analyses, confirm that the following items are present in the figure legend, table legend, main text, or Methods section.

n/a	Confirmed	
☐	☒	The exact sample size (*n*) for each experimental group/condition, given as a discrete number and unit of measurement
☐	☒	A statement on whether measurements were taken from distinct samples or whether the same sample was measured repeatedly
☐	☒	The statistical test(s) used AND whether they are one- or two-sided<br>*Only common tests should be described solely by name; describe more complex techniques in the Methods section.*
☒	☐	A description of all covariates tested
☐	☒	A description of any assumptions or corrections, such as tests of normality and adjustment for multiple comparisons
☐	☒	A full description of the statistical parameters including central tendency (e.g. means) or other basic estimates (e.g. regression coefficient) AND variation (e.g. standard deviation) or associated estimates of uncertainty (e.g. confidence intervals)
☐	☒	For null hypothesis testing, the test statistic (e.g. *F*, *t*, *r*) with confidence intervals, effect sizes, degrees of freedom and *P* value noted<br>*Give P values as exact values whenever suitable.*
☒	☐	For Bayesian analysis, information on the choice of priors and Markov chain Monte Carlo settings
☒	☐	For hierarchical and complex designs, identification of the appropriate level for tests and full reporting of outcomes
☒	☐	Estimates of effect sizes (e.g. Cohen's *d*, Pearson's *r*), indicating how they were calculated

Our web collection on statistics for biologists contains articles on many of the points above.

Software and code

Policy information about availability of computer code

Data collection	No unique software was used for data collection.
Data analysis	Data were analyzed with commercially available and open-source programs as stated in the methods section. Descriptive statistics were calculated with GraphPad Prism v8.0. Statistical tests were performed with SAS v9.4. Intracellular staining data were analyzed using FlowJo v10.8.1.

For manuscripts utilizing custom algorithms or software that are central to the research but not yet described in published literature, software must be made available to editors and reviewers. We strongly encourage code deposition in a community repository (e.g. GitHub). See the Nature Portfolio guidelines for submitting code & software for further information.

Data

Policy information about availability of data

All manuscripts must include a data availability statement. This statement should provide the following information, where applicable:

- Accession codes, unique identifiers, or web links for publicly available datasets
- A description of any restrictions on data availability
- For clinical datasets or third party data, please ensure that the statement adheres to our policy

The authors declare that the data supporting the findings of this study are available within the main and supplemental figures. All data is available from the corresponding author upon reasonable request. Source data are provided with this paper.

Human research participants

Policy information about studies involving human research participants and Sex and Gender in Research.

Reporting on sex and gender	NA
Population characteristics	NA
Recruitment	NA
Ethics oversight	NA

Note that full information on the approval of the study protocol must also be provided in the manuscript.

Field-specific reporting

Please select the one below that is the best fit for your research. If you are not sure, read the appropriate sections before making your selection.

☒ Life sciences ☐ Behavioural & social sciences ☐ Ecological, evolutionary & environmental sciences

For a reference copy of the document with all sections, see nature.com/documents/nr-reporting-summary-flat.pdf

Life sciences study design

All studies must disclose on these points even when the disclosure is negative.

Sample size	We used groups of 5 macaques, as n=5 is the minimal sample number for Wilcoxon rank sum exact test.
Data exclusions	No data were excluded.
Replication	Each binding study was repeated to confirm results. Within binding studies we tested multiple dilutions of antibodies to confirm the binding magnitude instead of relying on single data points. Neutralization assays have been validated to be reproducible and group geometric means are shown to identify the group trend. Binding assays, blocking assays and neutralization assays were repeated in 2-3 months using freshly thawed serum/plasma samples for each repeat. All attempt replication were successful.
Randomization	Macaques were distributed in groups to balance age, gender and weight whenever possible. Mice and other samples were distributed randomly.
Blinding	Neutralization, binding, and competition assays were performed by laboratories independent from the discovery laboratory. No other data was supplied until after the assay was complete. Statistics were not calculated until the study was complete, and were done so by statisticians independent from the discovery researchers.

Reporting for specific materials, systems and methods

We require information from authors about some types of materials, experimental systems and methods used in many studies. Here, indicate whether each material, system or method listed is relevant to your study. If you are not sure if a list item applies to your research, read the appropriate section before selecting a response.

Materials & experimental systems

n/a	Involved in the study	
☐	☒	Antibodies
☐	☒	Eukaryotic cell lines
☒	☐	Palaeontology and archaeology
☐	☒	Animals and other organisms
☒	☐	Clinical data
☒	☐	Dual use research of concern

Methods

n/a	Involved in the study	
☒	☐	ChIP-seq
☒	☐	Flow cytometry
☒	☐	MRI-based neuroimaging

Antibodies

Antibodies used	DH1041, Li D, et al. Cell. 2021. PMID: 34242577<br>DH1047, Li D, et al. Cell. 2021. PMID: 34242577

DH1050.1, Li D, et al. Cell. 2021. PMID: 34242577
SARS-CoV-2 Nucleocapsid antibody, 1:2000, GeneTex, Catalog # GTX135357
anti-CD4 APC-H7 (BD Biosciences), anti-CD95 PE (BD Biosciences), anti-CD28 PerCP-Cy5.5 (BD Biosciences;), and anti-CCR7 FITC (R&D Systems). After fixation and permeabilization with Cytofix/Cytoperm solution (BD Biosciences), the cells were stained with anti-IFNγ PECy7 (BD Biosciences), anti-TNFα AF700 (BD Biosciences), anti-IL2 APC (BD Biosciences), anti-CD3 Pacific Blue (BD Biosciences), and anti-CD69 ECD4+ (Beckman Coulter).

Validation

Validation of each antibody is performed prior to use by the flow cytometry core.

Eukaryotic cell lines

Policy information about cell lines and Sex and Gender in Research

Cell line source(s)

Vero E6 cells and HEK 293T/17 cells were from ATCC. FreeStyle 293-F cells are from Thermo Fisher. 293T/ACE2.MF cells were kindly provided by Drs. Mike Farzan and Huihui Mu at Scripps.

Authentication

Each cell line is provided with a certificate of analysis. Cell identity is verified by morphology or fluorescent markers expressed.

Mycoplasma contamination

All cell lines undergo mycoplasma testing every 60 days. All cells tested negative for mycoplasma contamination.

Commonly misidentified lines
(See ICLAC register)

None to report.

Animals and other research organisms

Policy information about studies involving animals; ARRIVE guidelines recommended for reporting animal research, and Sex and Gender in Research

Laboratory animals

Cynomolgus macaques. Cynomolgus macaques were on average 8-9 years old and ranged from 2.75 to 8 kg in body weight. Male and female macaques per group were balanced when availability permitted. The study protocol and all veterinarian procedures were approved by the Bioqual IACUC per a memorandum of understanding with the Duke IACUC, and were performed based on standard operating procedures. Macaques studied were housed and maintained in an Association for Assessment and Accreditation of Laboratory Animal Care-accredited institution in accordance with the principles of the National Institutes of Health. All studies were carried out in strict accordance with the recommendations in the Guide for the Care and Use of Laboratory Animals of the National Institutes of Health in BIOQUAL (Rockville, MD). BIOQUAL is fully accredited by AAALAC and through OLAW, Assurance Number A-3086. All physical procedures associated with this work were done under anesthesia to minimize pain and distress in accordance with the recommendations of the Weatherall report, "The use of non-human primates in research." Teklad 5038 Primate Diet was provided once daily by animal size and weight. The diet was supplemented with fresh fruit and vegetables. Fresh water was given ad libitum. All monkeys were maintained in accordance with the Guide for the Care and Use of Laboratory Animals.

Eleven-month-old female BALB/c mice were purchased from Envigo (#047) and were used for the SARS-CoV, SARS-CoV-2 WA-1, SARS-CoV-2 B.1.351, and RsSHC014-CoV protection experiments. The study was carried out in accordance with the recommendations for care and use of animals by the Office of Laboratory Animal Welfare (OLAW), National Institutes of Health and the Institutional Animal Care and Use Committee (IACUC) of University of North Carolina (UNC permit no. A-3410-01). Animals were housed in groups of five and fed standard chow diets. Virus inoculations were performed under anesthesia and all efforts were made to minimize animal suffering.

Wild animals

The study did not involve wild animals.

Reporting on sex

Males and females were used in the studies.

Field-collected samples

The study did not involve collection of samples from animals in the field.

Ethics oversight

The macaque studies were performed at Bioqual. Prior to study commencement, all procedures and materials to be used in the study were approved by the Bioqual IACUC (Assurance Number A-3086). The Duke University Institutional Biosafety Committee approved protocols for research involving recombinant DNA.

The mouse study was carried out in accordance with the recommendations for care and use of animals by the Office of Laboratory Animal Welfare (OLAW), National Institutes of Health and the Institutional Animal Care and Use Committee (IACUC) of University of North Carolina (UNC permit no. A-3410-01).

Note that full information on the approval of the study protocol must also be provided in the manuscript.
